# Overcoming resistant cancerous tumors through combined photodynamic and immunotherapy (photoimmunotherapy)

**DOI:** 10.3389/fimmu.2025.1633953

**Published:** 2025-07-17

**Authors:** Glory Kah, Heidi Abrahamse

**Affiliations:** Laser Research Centre, University of Johannesburg, Doornfontein, Johannesburg, South Africa

**Keywords:** cancer, resistant, photodynamic therapy, immunotherapy, photoimmunotherapy, nano-photoimmunotherapy

## Abstract

Cancer is a major health problem as it causes significant mortality globally. In the last decades, conventional and recent therapeutic approaches have been used in oncology for cancer treatment. Despite this, the complete eradication of cancer is challenging, as the existing therapeutic strategies for cancer are typically faced with limitations. This is linked to cancer resistance to treatment, which arises because of the versatile nature of cancerous cells. Novel anticancer therapeutic procedures based on immune system activation, such as photodynamic therapy (PDT) and immunotherapy (IOT), are promising in treating resistant tumors. PDT is a minimally invasive treatment that induces cellular reactive oxygen species (ROS) production for direct elimination of cancerous cells, but can also trigger anticancer effects by activating the immune system of the host. IOT also has significant anticancer efficacy and has emerged as an advanced anticancer treatment that mainly enhances and stimulates the innate immune system of the body to identify and destroy cancerous cells. IOT can also instigate a long-lasting anticancer response by harnessing the body’s immune system. PDT and IOT, when used alone, cannot tackle the issue of cancer resistance. This review elucidates the principles, benefits, and setbacks of PDT and IOT, along with the unique attributes that render them suitable for cancer combination therapy. It underscores the advancement of cancer PDT when utilized in combination with IOT (photoimmunotherapy), while also encapsulating the preclinical evidence regarding the efficacy of photoimmunotherapy, and its combination with nanotechnology (Nano-photoimmunotherapy). The key findings indicate that photoimmunotherapy preclinical methods hold great promise in cancer treatment, as they can directly destroy cancer cells through PDT while also stimulating an increased anticancer immunity through co-delivery of IOT agents. Target-specific moieties can be used in nanotechnology-based anticancer photoimmunotherapy techniques to get past resistance and other therapeutic obstacles. However, clinical utilization of photoimmunotherapy procedures is greatly required to warrant the full efficacy.

## 1 Introduction

Cancer is a serious global health concern, as it is a great cause of mortality globally. It is accountable for about one in six deaths globally and with about 10 million deaths annually. Cancer is a transversal disease that affects both developed and emerging countries across different ethnicities and cultures (1, 2). Even though, an excessive burden is shouldered by the low and middle-income nations as a result of restricted access to early diagnosis and treatment, and increased exposure to risk factors including, unhealthy diets, poor physical activity, tobacco use, and some infections such as hepatitis B and HPV (2). Various treatment strategies are developed for cancer intervention, including surgery, radiotherapy, and chemotherapy (conventional therapy). Other recent strategies used in cancer treatment include targeted therapy, immunotherapy, stem cell therapy, ablation therapy, sonodynamic therapy, chemodynamic therapy, ferroptosis-based therapy, and radionics (3, 4). These modern cancer therapeutic procedures have significantly improved cancer treatment over the past 30 years (5); however, cancer treatment failure is still widespread due to the ineffectiveness of these therapies (6). This is linked to cancer resistance to monotherapies (5). The phenomenon of cancer resistance to treatment commonly arises in clinical practice, thus resulting in poor patient survival. Also, cancerous cells with resistant attributes frequently show cross-resistance to different anticancer treatments or drugs that may not be structurally relevant, and this has been attributed to the appellation multidrug resistance (MDR). The MDR phenomenon is a major impeding obstacle to treatment success, with consequential impacts like cancer recurrence and cancer-related death (7).

Cancer treatment resistance can be grouped into acquired and intrinsic resistance depending on the time the resistance arises. Intrinsic cancer resistance is recognized as the primary resistance that originates from endogenous factors existing in tumor cells before any treatment applications. These factors give cancerous cells survival benefits and the potential to adapt to stress from primary therapy (8, 9). On the other hand, cancer-acquired drug resistance is typically mediated by adaptive changes that antagonize cancerous cells’ susceptibility to an administered treatment, thus reducing the treatment’s efficacy (7, 9).

The reduced response of cancerous cells to treatment is also linked to different mechanisms, most often involving the co-action of genetic and non-genetic factors. Tumor cell genetic factors are identified as major contributors to treatment resistance. These genetic factors include oncogene amplification in bypass or compensatory pathways, acquired drug target mutations, genetic diversity, and changes in epigenetics, which can also affect DNA repair, tumor cell plasticity, intratumor heterogeneity, and tumor cell susceptibility to pathways leading to cell death, hence resulting in multifactor-mediated resistance (7, 10). Yet, cancer drug resistance has been identified in which there is no genetic mutation in patients with various types of cancers (10, 11). The genotype alterations can be independent of the phenotype changes when the resistance is mediated via the metabolic inactivation of cancerous drugs, drug compartmentation, fewer intracellular transporters of drug concentrations, and reversible transcriptional or posttranslational controls on adaptive pathways induced by the drugs (7).

However, combination cancer treatments are stated to increase the likelihood and strength of treatment responses while lowering the probability of treatment resistance being developed in the patient (12). The cornerstone of cancer combination therapy is to target pathways that perpetuate or cause cancer precisely. Combination therapy often works in an additive or synergistic manner, thus requiring a reduced dose of each separate drug. Combination therapy can offer a toxic effect on cancerous cells while preventing damage to healthy cells. This can be materialized if one of the utilized drugs is cytotoxically antagonistic to a different drug within normal cells, thus shielding healthy cells from cytotoxic damage (13). An effective cancer combination therapy might overcome the shortcomings of conventional mono-therapeutic treatment, such as the non-selective targeting of active proliferating cells, which eventually results in the death of both malignant and healthy cells. Monotherapy, like chemotherapy, damages both healthy and cancerous proliferating cells, causing several hazardous effects. It can significantly weaken the immune system of patients by attacking cells in the bone marrow and amplifying the patient’s susceptibility to diseases (13, 14).

Nonetheless, in the battle against tumors that are resistant to conventional therapy, novel anticancer therapy based on immune system activation is encouraged. Such therapy includes photodynamic therapy (PDT) and immunotherapy (IOT) (15). An anticancer combination therapy that explores PDT and IOT (photoimmunotherapy) may overcome the issues of cancer resistance since this type of combination therapy is documented to prevent tumor metastasis, activate the immune system’s memory cells, and stop the recurrence of tumors (16, 17). Besides, photoimmunotherapy in combination with advanced nanotechnology (nano-photoimmunotherapy) is noted to offer a better therapeutic efficacy against resistant cancer (18). In this review, we describe PDT and IOT, highlighting their significance in anticancer immune stimulation. The preclinical evidence on photoimmunotherapy and nano-photoimmunotherapy in combating cancer resistance is also discussed.

## 2 Photodynamic therapy

PDT is a photochemical therapeutic procedure that utilizes laser irradiation at a defined wavelength to stimulate the transfer of photoelectrons to nearby oxygen molecules, thereby generating singlet oxygen, which is lethal to cancerous cells (19). This therapeutic procedure is less invasive, suitable for treating squamous cell carcinoma, and offers good spatiotemporal selectivity (20). Apart from being directly lethal to cancerous cells, PDT can stimulate inflammatory reactions that encourage the creation of tumor-associated antigens from the remnants of cancerous cells, leading to immunogenic cell death (21).

### 2.1 Principle of photodynamic therapy

**Figure 1:**
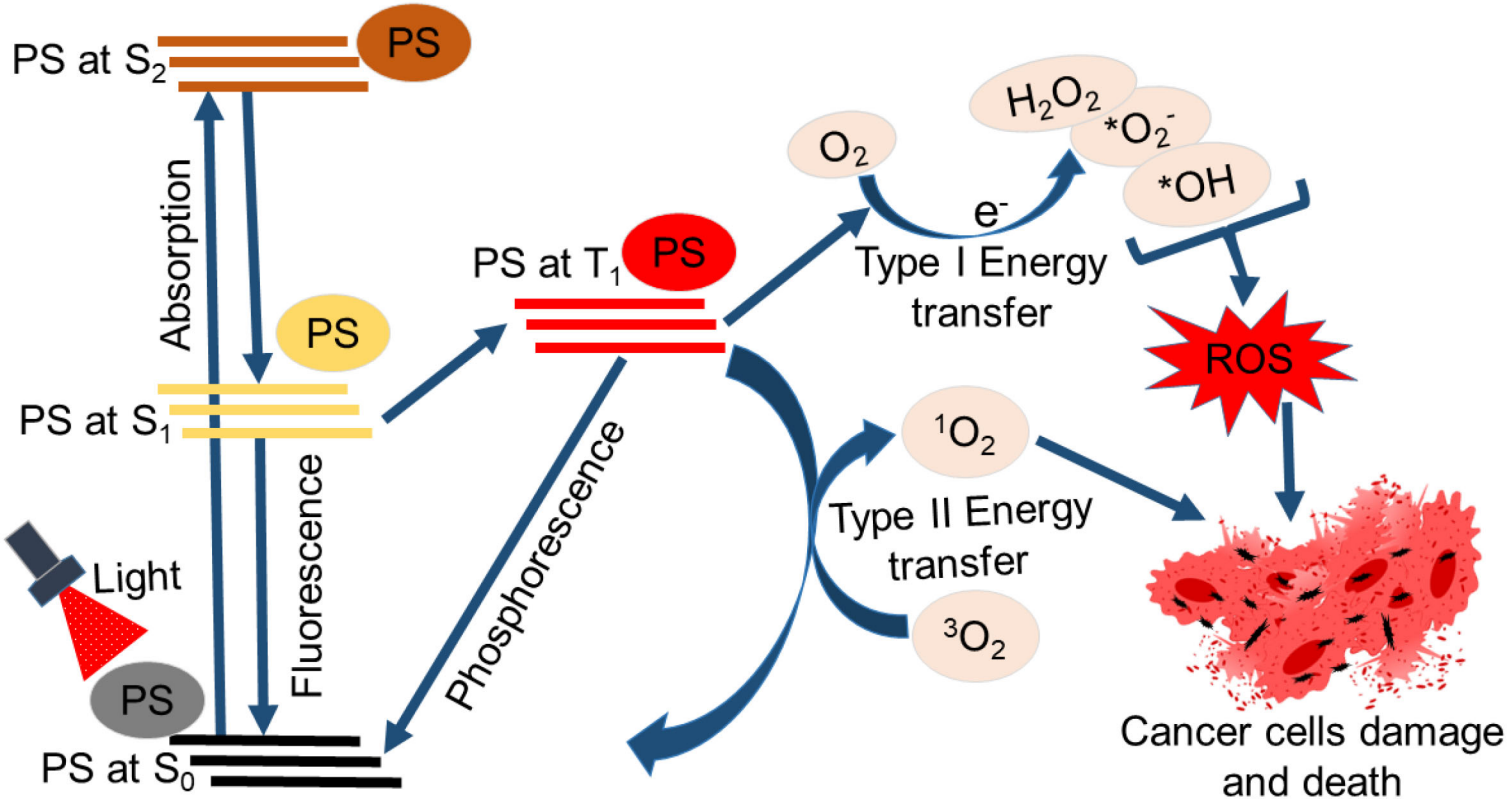
Schematic representation of photodynamic therapy’s principle. The absorption of light by the photosensitizer (PS) excites it from the ground state (S_0_) to a higher energy orbital (S_1_ or S_2_) excited state. The PS returns to the original S_0_ or excites to the triplet state (T1). Type I reaction (Type I energy transfer) occurs where the PS at T1 reacts with oxygen, leading to ROS production. Type II reaction (Type II energy transfer) may also occur when the PS at T1 reacts directly with oxygen in its ground state (³O_2_) by transferring energy to generate singlet oxygen (¹O_2_). The ROS generated induces damage to cancerous cells and death.

Light irradiation of photosensitizer (PS) stimulates photon absorption and the excitation of the PS to the singlet state (S_1_), where there is a shift of electrons to an orbital with higher energy (**Figure 1**). At this state (a short-lived state and typically not stable), the PS may go back to the ground state (S_0_) through the conversion of its energy to fluorescence or heat. This feature is so handy for applications in monitoring and diagnostic procedures (22). Alternatively, intersystem crossing may happen, resulting in the PS being excited to a triplet state (T_1_). The PS in the T_1_ can convey energy via phosphorescence or collide with different molecules, creating reactive chemical species through two kinds of molecular reactions. T_1_ PS can also react with different types of solvents or organic substrates to transfer a single proton or electron, forming cation species or radical anions, respectively. Mostly, reactions of the PS with electron donor substrate occur, forming PS that eventually reacts with oxygen, generating radical superoxide anion. This is classified as a type I reaction. Yet, a type II reaction could take place if the PS at T_1_ directly reacts with oxygen in its ground state (^3^O_2_) by transferring energy to generate singlet oxygen (^1^O_2_), noted as reactive oxygen species (ROS) (22, 23). The molecular products of PDT, such as superoxide anions and singlet oxygen, will promote cytotoxicity since both products can even react directly with biomolecules like nucleic acids, proteins, and lipids, leading to their degradation (24). The superoxide anions that are created from type I reactions are non-damaging, especially in biological systems. Despite that, these superoxide anions can participate in reactions that generate hydrogen peroxide. A Fenton reaction may also occur where the superoxide anion instead reacts with hydrogen peroxide, forming highly reactive hydroxyl radicals. These radicals are ultimately capable of reducing hydrogen atoms in biomolecules or adding to the side of biomolecules with double bonds. For example, reactions of fatty acids with hydroxyl radicals can generate a hydroxylated product, which is also a radical, thus originating a reaction chain involved in lipid peroxidation and consequently causing damage to the cell (22, 25). Moreover, ROS can directly damage lipids, proteins, and DNA in cancerous cells, causing alterations in ion transportation and cellular metabolism and an imbalance in homeostasis. The mitogen-activated protein kinase is stimulated in response to induced oxidative stress. Also, numerous cytokines and mediators involved in cell death processes such as autophagy, necrosis, and/or apoptosis are released. These processes or pathways could occur simultaneously and are not mutually exclusive with the same cell population. Different important parameters could contribute to the occurrence of a particular type of cell death pathway. These parameters include drug dose, PS intracellular distribution, light dose (illumination total time and fluence rate), the cell type being investigated, and the available amount of oxygen (15). Cell death following PDT activation is typically induced via autophagy, necrosis, and/or apoptosis (26–28). However, some newly identified cell death processes are linked with PDT outcomes, including paraptosis (typified with elevated cytoplasmic vacuolization that does not involve nuclear fragmentation or caspase activation) (27, 29), pyroptosis (inflammation-induced cell death) (30), ferroptosis (iron-dependent lipid peroxidation-driven cell death) (31), and necroptosis (controlled cell death mimicking attributes of necrosis and apoptosis) (32). These cell death pathways or programs can each result in the release of damage-associated molecular patterns (DAMPs) or alarmins. The pattern recognition receptors (PRRs) on immune cells are able to recognize DAMPs. Binding of DAMPs to PRRs activates the immune cell and thus immunogenic cell death (ICD) (26).

### 2.2 Photodynamic therapy photosensitizers, localization, and dose to induce ICD

Through the years, photosensitizers (PSs) have been modified to address the setbacks of their predecessors and to enhance the therapeutic efficacy of PDT. This has led to PSs being grouped or divided into various generations, including first, second, third, and even fourth generations of PDT PSs. The first-generation PSs are often natural compounds and hematoporphyrin primary derivatives like porfimer sodium (Photofrin) (33, 34). These PSs are faced with limitations such as poor tissue penetration, being prone to photobleaching, exhibiting poor solubility due to their hydrophobic nature, and long cutaneous photosensitivity (33, 35). The second-generation PSs are designed to solve these limitations. Second-generation PSs can provide decreased skin photosensitivity, reduced tissue accumulation periods, and enhanced light absorption at wavelengths from 650 to 800 nm (36, 37). On the other hand, the third-generation PSs are developed for specific tumor targeting, improving the selectivity and efficacy in intracellular delivery and multimodal therapeutic applications. They can be obtained via the conjugation of second-generation PSs with the targeted molecules or encapsulated in nanomaterials (33, 36). Specifically, the incorporation or conjugation of PSs with nanomaterials has paved the way for their utilization as nanomedicine. The fourth-generation PSs are designed by making use of porous delivery systems, such as metal–organic frameworks and mesoporous silica (34). They are generally considered to provide advanced targeting modes of action or combine many therapeutic modalities (36).

PSs can accumulate to initiate their damaging effects on various cell compartments based on the chemical attributes of the PS. The PS subcellular localization involves various organelles, including mitochondria, endoplasmic reticulum (ER), lysosomes, Golgi apparatus, nucleus, and plasma membrane (38–40). PS localization is vital in determining if the cell death following PDT will be triggered via an immune response. An important prerequisite for ICD is the formation of ROS in the ER. This induces oxidative stress, after which one of the major DAMPs [calreticulin (CRT)] is exposed, causing the activation of the host immune system to fight cancer (26, 41). This is indicative that a successful cancer eradication strategy via PDT-induced ICD will logically require the targeting of the PS within the ER. Studies have established that direct accumulation of hypericin in the ER leads to elevated ROS production and the development of a robust immune response following PDT (41, 42). But not all PSs accumulate or build up in the ER. In order to accumulate in the ER, the PS must possess amphiphilic and hydrophobic characteristics. The PS’s charge also influences its ability to accumulate in the ER. Hydrophilic PSs are often found in the lysosomes/endosomes before being dispersed in the cytoplasm. When the PS is directly collected in the ER, the effectiveness of PDT and its immunogenic effects are both increased (41).

However, using PS localized in other cell compartments for PDT might also display immunogenic properties. For instance, PDT immunogenicity was established following the localization of PS in the lysosomes. The immunogenicity was exhibited in mouse fibrosarcoma (MCA205 murine prophylactic tumor vaccination mode) via dendritic cell (DC) maturation, release of DAMPs, and an effective reduction in tumor growth (43). However, a PS can be localized in multiple cellular compartments simultaneously (40, 44). Interestingly, two PSs used at the same time target and harm two cellular compartments simultaneously. For instance, studies confirm the targeting of the mitochondria and the lysosome at the same time using benzoporphyrin derivative (BPD, verteporfin) and N-aspartyl chlorin E6 (NPe6) or photofrin, respectively. This PDT technique sequentially induced photodamage starting from the lysosomal, then to the mitochondrial, leading to higher tumor eradication than using a single PS (45–47). Despite that, it was not clarified whether this method could activate an ICD. Still, the administration of two PSs sequentially to target distinct subcellular compartments might be an encouraging strategy to induce ICD, as more intriguing discoveries may be anticipated.

The administered dose of the PS also plays an instrumental role in the overall treatment outcome. High doses of PS can result in aggregation-induced quenching which causes a reduction in the PS optical properties. The systemic administration of such a high dose of PS could cause abnormal accumulation and distribution, resulting in a phototoxic effect (48). Also, high PS dosage raises the risk of adverse effects such as non-scarring skin lesions, erythema, pain, and the death of healthy cells around the area exposed to light (49, 50). It is therefore crucial for an ideal PS to be chosen for PDT, and this PS should trigger an ICD with the least amount of damage to healthy cells. The PS can also infiltrate healthy cells, so elevated PS doses can promote severe dark toxicity to healthy cells. This can harm different cell types, especially cells in the brain, since morphofunctional abnormalities such as those in the neuron-glial network can cause a drastic malfunction in the central nervous system and exacerbate the patient’s situation (51, 52). Recent PDT techniques indicate that elevated doses of PS could be circumvented by employing advanced nanostructure delivery systems. The delivery system can help deposit the PS in the targeted cellular compartment in the tumor tissue, leading to the stimulation of ICD while limiting exposure to healthy cells.

### 2.3 PDT damage, inflammation, and immune response against cancer

Tumor damage induced by PDT commonly involves the following: 1) ROS stimulating the direct killing of tumor cells by apoptosis, autophagy, and necrosis. 2) The vascular system targeted by PDT PSs forms thrombi, leading to tumor tissue hypoxic infarction. 3) An inflammatory response can occur, resulting in an antitumor immune response, and is triggered by inflammatory substances that are released by the tumor cells that undergo apoptosis or necrosis (6, 53). It is important to note that the PDT processes, including the cell cycle arrest, autophagy, and apoptosis, may all happen at the same time following a single session of PDT treatment. Sasnauskiene et al. findings reveal that there is a dose-dependent correlation between the amount of cellular damage via oxidative stress. Elevated cell cycle arrest and autophagy, but without apoptosis were confirmed when the cellular toxic dose was augmented to 50% (54). Nonetheless, when the toxic dose was above 70%, the cells exhibited substantial cell cycle arrest, autophagy, and apoptosis. PDT-induced damage to the blood vessels principally depends on tumor tissue attributes of having large vascular gaps and poor integrity, thus promoting the aggregation of PS (55). The enrichment of the vascular endothelial tumor cells with PS following PDT photoactivation causes numerous physiological reactions, such as vasoconstriction and platelet aggregation. This results in ischemia, tumor vascular blockage, and hypoxia (6, 56). Moreover, different types of white blood cells, such as dendritic cells (DCs), macrophages, and neutrophils, are recruited thanks to the direct ablation action of PDT against tumor cells, which also liberate inflammatory mediators. The white blood cells subsequently trigger further tumor suppression via the stimulation of the immune cascade (6, 57).

PDT can also stimulate the interaction between the immune system of the adaptive and the innate arm (58, 59). Tumor microenvironment (TME) changes may occur by inducing the expression of mediators (acute-phase response mediators) and pro-inflammatory in the area irradiated. This could lead to a cascade process that induces systemic inflammation and adaptive and innate immunity. As a restricted treatment, PDT indirectly promotes the initiation of an acute inflammatory response while also directly damaging the tumor. PDT-induced oxidative stress in tumor cells can boost the release of inflammatory cytokines and inflammatory transcription factors, as well as improve the distribution of heat shock proteins (HSPs) (58). The invasion of the tumor site by leukocytes leads to their production of cytokines and pro-inflammatory factors. The mechanism of PDT can give rise to a robust inflammatory response, promoting neutrophils to quickly move to the treatment site, resulting in enhanced immunity and tumor response rate. In addition to impacting the proliferation/survival of tumor T cells and directing the production of PDT antitumor immunity, neutrophils also directly interact with photodamaged cells and later on eliminate the tumor cells that are photodamaged (60). Findings from *in vivo* studies indicate that PDT neutrophils bind and cluster on the microvascular wall. This provides supporting information that correlates antitumor response with neutrophil activity (61). Nonetheless, complement system activation has emerged as an antitumor mediator, and it also raises secondary inflammatory mediators, including histamine, coagulation factors, thromboxane, leukotrienes, and cytokines (such as IL-1β, IL-6, IL-10, G-CSF, and TNF-α). Complement activation produces transmembrane channels, damages the plasma membrane integrity, and results in lysis and cell death. Besides the complement cascade system, natural killer cells, phagocytes [neutrophils, macrophages, and DCs], and cellular elements are all components of the innate immune system response. The activation of the complement and innate immune system, as well as the cytokine activity, all function together to activate the innate immune system in responding to PDT (6, 62, 63).

### 2.4 Photodynamic therapy in immunogenic cell death and DAMPs

**Figure 2:**
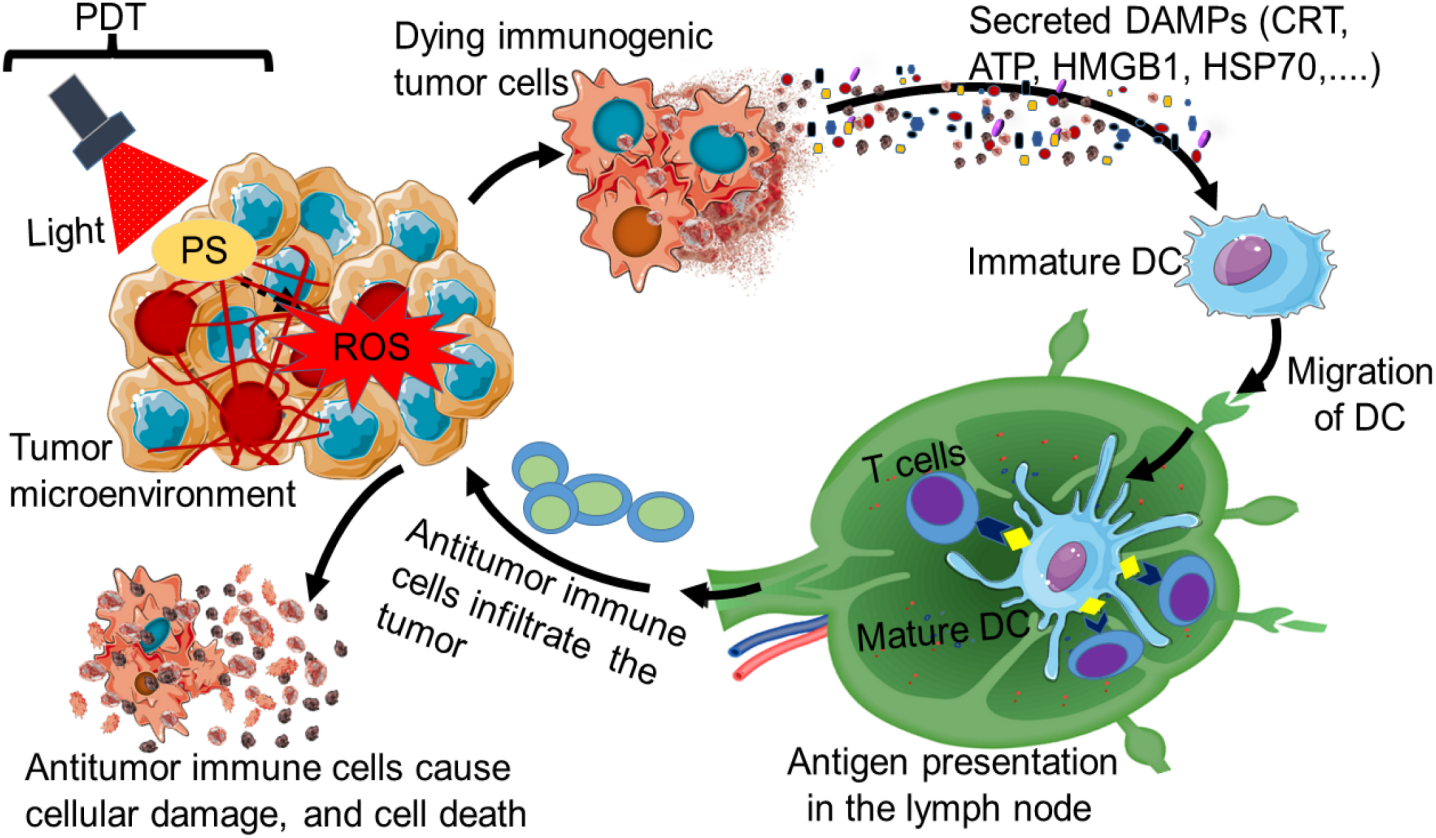
Immunogenic cell death induced by photodynamic therapy (PDT). The stimulation of a PDT photosensitizer (PS) by light of a specific wavelength can lead to immunogenic cell death (ICD). The dying immunogenic tumor cell can secrete on its surface various types of damage-associated molecular patterns (DAMPs) such as CRT, ATP, HMGB, and HSP70. The secreted DAMPs can promote the recruitment of antigen-presenting cells, especially dendritic cells (DCs). The DCs can migrate to the lymph node, where they mature for antigenic presentation to T cells. This results in the induction of antitumor immunity, where activated antitumor immune cells from the lymph node move and infiltrate the tumor to cause cellular damage and death.

Cancer PDT immune response leads to ICD of cancerous cells by inducing the liberation of tumor-related antigens from cancerous cells’ remnants. It can also further excite the activation, infiltration, and proliferation of antigen-specific T lymphocytes (64). ICD is also characterized by a specific response mechanism that causes cellular and organellar stress, ultimately leading to cell death and the exposure, passive secretion, or active release of several DAMPs as presented in **Figure 2**.

DAMPs are spatiotemporally presented during ICD and are recognized by a unique type PRR found on antigen-presenting cells, starting a cascade of reactions that can trigger adaptive and innate immunologic responses (65, 66). DAMPs released by dying cells during ICD include the cytoplasmic protein annexin A1 (ANX1), the non-histone chromatin-binding protein high-mobility group box 1 (HMGB1), endoplasmic reticulum (ER) chaperones [like heat-shock proteins (HSPs) and calreticulin (CRT)], as well as interferons (IFNs) (*de novo* synthesized type I IFNs) and adenosine triphosphate (ATP) (67–69). The recognition of DAMPs by the PRRs expressed in immunogenic adaptive and innate cells leads to effector cells’ chemoattraction, stimulation, maturation, and/or homing. These processes work together to suppress the tumor (65).

Studies indicated that oxidative stress induced by PDT treatment can trigger the production of DAMPs and tumor antigens, resulting in antitumor immunity (64). The stimulation of DAMPs following PDT-treated cancerous cells is widely documented. Nonetheless, the DAMPs pattern may differ depending on the cancerous cell type and the treatment regimen (38). Moreover, the production of DAMPs following cell death has become a crucial component of the network of intercellular communication, as it influences both inflammatory processes and immunological responses (70). The PDT-stimulated DAMP samples after PDT were noted to have DAMPs such as high mobility group box 1 (HMGB1), HSPs (HSP60, HSP70, HSP90), CRT, and ATP (64). **Table 1** presents the various types of DAMP that could be involved in ICD. Also, HSPs such as HSP34, HSP27, and HSP72/73 have been induced by PDT (38). The DAMPs are upregulated and translocated to the membrane, where they might be recognized and eliminated by the cells of the innate immunity as a danger sign, thus stimulating an innate immune response (62). It is reported that following PDT treatment, immunocompetent mice’s tumor response was improved by CRT (DAMP molecule) as opposed to immunodeficient mice (78).

**Table 1:** Various types of DAMP that can be produced following cancer PDT treatment.

DAMPs	Description and function	Responder cells	Reference
Heat Shock Proteins (HSPs) like HSP60, HSP70, HSP90, gp96, GRP94, GRP78	HSPs, or stress proteins, are often found in organelles or intracellular areas. They are produced by the cells in reaction to a variety of stress stimuli, like ultraviolet light. However, they are displayed on the surface of damaged or dying cells and are a key component in immunomodulatory processes. For instance, it has been discovered that surface-exposed HSP90 and HSP70 impact antigen processing/presentation and phagocytosis. Immunogenicity through dying cells is noted to be defined by HSP90. Though HSPs can be actively released through the non-classical secretory route, HSPs are more frequently secreted passively by dying cells. Vital tumor antigens can be carried by secreted HSPs, facilitating the appropriate uptake and processing of antigens by antigen-presenting cells. Moreover, they can activate immune cells to secrete different types of pro-inflammatory cytokines. Recent evidence indicates that extracellular HSP90b can hinder the stimulation of latent TGF-b1.	Monocytes, neutrophils	(59, 71, 72)
High mobility group box-1 (HMGB1)	They are nuclear chromatin-binding proteins that can act as a DAMP molecule at the exterior of cells and as a nuclear protein when inside the nucleus. It is noted to have strong cytokine-like characteristics, and when HMGB1 is produced by dying cells, it activates immune cells to release a variety of pro-inflammatory cytokines.	Monocytes, neutrophils	(59, 71, 72)
Calreticulin (CRT)	They are multifunctional proteins that are usually present in different intracellular organelles/regions (especially in the ER) (2, 13). Under stress conditions (commonly stress in the ER), their levels are increased extracellularly (exo-CRT) on the plasma membrane. They function as a danger “eat me” signal on the plasma membrane as they enhance the immunogenic ability of dying cells.		(59, 71–73)
Phosphatidylserine (PtdSer)	Phosphatidylserine can move from the cell’s inner to the outer leaflet (compartment) if the cell is injured or dying to function as an “eat me signal” to mediate anti-inflammatory responses and effective phagocytosis, thanks to its interaction with multiple receptors on immune cells. They can also interact with opsonins (such as growth arrest-specific gene 6 (Gas6), β2-glycoprotein (β2GP1), milk fat globule EGF/factor VIIC (MFG-E8), and Annexin-V).	Macrophage, dendritic cells	(71, 72, 74, 75)
Adenosine triphosphate (ATP)	They are often intracellular high-energy molecules, yet they can be secreted under specific stress conditions by apoptotic and necrotic cells. It is also possible for ATP generated extracellularly to help in immune cell chemoattraction.	Dendritic cells, microglia	(71, 72, 75)
Covalent/Cross-linked dimer of ribosomal protein S19 (dRP S19)	Description and function: They are small ribosomal subunit constituents and can be secreted by necrotic cells. They can attract different immune cells by acting as a chemotactic factor.	Monocytes, neutrophils	(59, 71, 72)
Calgranulin family members S100S (S100A8, S100A9, S100A12)	They are calcium-binding proteins expressed by different cell types. When calgranulins function as “find me signals” by attracting different immune cells. They can activate the pro-inflammatory cytokines, thanks to their interaction with the receptor (TLR4/RAGE) on immune cells.	Monocytes, neutrophils	(59, 71, 72)
Uric acid or Monosodium urate	The intracellular uric acid stockpiles in dying or ischemic cells are released. Also, more uric acid is produced after cell death as a result of the enzymatic degradation of nucleic acids.	Dendritic cells, neutrophils, CD4+ and CD8+ T cells	(59, 72, 76)
Spliceosome-associated protein 130 (SAP130)	SAP130 is a histone deacetylase complex subunit and can be produced by dying cells following apoptosis and regulated necrosis.	Macrophages	(59, 72, 77)

A typical characteristic of dying PDT-treated cancerous cells is the plasma membrane surface exposure of the calcium-binding protein CRT. Normally, CRTs are located in the lumen. Yet, when CRTs are externally exposed, they can be recognized by lipoprotein receptor-related protein 1 (LPR1, CD91) of low density to provide an ‘eat me’ type of signal to antigen-presenting cells. ER stress is linked with CRT exposure, which results from misfolded proteins piling up, thus causing an unfolded protein response (38). However, the mechanisms for CRT induced by PDT can vary based on PS type. For instance, treatment with Rose Bengal acetate led to CRT exposure and the co-translocation of the ER protein (79, 80), while the co-translocation of the ER protein was not noted in hypericin-based mediated PDT (41, 41). In addition, the eukaryotic initiation factor 2α phosphorylation is widely considered to be needed for CTR exposure and is vital for UPR induction (81). Yet in a hypericin-based PDT, this phosphorylation did not occur (41, 41). Also, macrophage activation can cause the release of CRT, where it may bind to viable cell surfaces and, in doing so, promote their clearance (82, 83). PDT-damaged cells found bound by CRT, were noted to activate migration, macrophages, and phagocytosis. Also, the host immune cells, including neutrophils, macrophages, and DCs, can be activated by DAMPs. DAMP expression induced thanks to PDT treatment can be a powerful immune response responsible for tumor ICD (6).

DAMPs are necessary for tumor-associated antigens presentation to antigen-presenting cells, which helps to stimulate an immune response against cancerous cells. Dead/dying PDT-treated cells are reported to release PDT-related DAMPs such as ATP and HMGB1 (43, 84, 85). Photosens and photodithazine have recently been reported to induce ICD which, was associated with the emission of HMGB1 and ATP (43). HMGB1 can trigger a response to innate immunity by interacting with 2 and 4 toll-like receptors and potentially with recognized receptors on antigen-presenting cells. ATP, on the other hand, encourages antigen-presenting cell recruitment by attaching to purinergic receptors, and this is read by antigen-presenting cells as a signal to ‘find me.’ ATP may be released actively from the cell, which is controlled by a particular signaling pathway, or released passively due to the loss of the integrity of the plasma membrane (86). Similar to the induced CRT mechanism, the exact mechanism stimulating ATP release following PDT appears to present unique attributes. For instance, hypericin-mediated PDT stimulated ATP secretion in a way that is autophagy-independent, which is contradictory to ICD stimulated by chemotherapy (87, 88).

### 2.5 Photodynamic therapy advantages and disadvantages

PDT has several advantages over conventional cancer therapeutic techniques. PDT has no long-term negative effect when correctly administered, yet first-generation PSs can induce modest, temporary photosensitivity in some areas, such as the eyes and skin (89, 90). When comparing PDT to surgical procedures, its adverse effects are frequently not severe, and the duration is not prolonged as in radiotherapy or chemotherapy. Also, PDT is typically administered as an outpatient procedure. Tumor mortality is substantially caused by the destruction of the tumor’s associated vasculature (91, 92). PDT’s dual selectivity allows it to be directly applied and accurately target a precise tissue (90). PDT can be administered repeatedly in the same treatment area if necessary, as opposed to being dispersed like in radiotherapy. Scarring after the PDT healing process is generally minimal. Moreover, PDT treatment typically costs less than the majority of cancer treatments (90, 93). PDT can also alter the microenvironments of the stroma and blood vessels, increasing their vulnerability to further treatments like IOT and chemotherapy (15).

Cancer tumors of the digestive tract, lung, lung lining (malignant pleural mesothelioma), head and neck, bladder, skin (basocellular carcinoma), and cervix have been treated using PDT (94). However, some disadvantages are linked with PDT. Tumor cells are documented to be resistant to PDT (95, 96). Also, resistance to PDT photosensitizers may develop via similar mechanisms as those with conventional drugs, such as decreased uptake, elevated inactivation of the drug, drug efflux, and alterations in intracellular trafficking. A combination treatment strategy with at least two distinct treatment plans is one potential tactic for conquering tumor resistance (94). Also, PDT is ineffective for disseminated metastases because current PDT methods do not deliver whole-body laser light treatment, and the PDT effect often occurs at the treated location (97). This affirms that PDT yields limited therapeutic results on distal and metastatic tumors (23, 98). The effect of PDT relies on tumor tissue oxygenation, and this is commonly impeded by the thick tumor masses or necrotic tissue (90). The consumption of oxygen during PDT exacerbates tumor hypoxia, causing a vicious circle. Hypoxia in tumor cells can enhance the growth of immunosuppressive cells such as M2-type macrophages, hindering antitumor immunity and ICD, subsequently leading to tumor progression and relapses (99, 100).

Treating deeply infiltrated or deep-seated tumors is challenging using PDT due to visible light’s (short light wavelength of about 400–700 nm) poor tissue penetration (101). Furthermore, PDT alone is not enough to trigger a substantial immune response since tumor cells produce immunosuppressive cytokines or other types of tumor-enhancing substances by a non-immunogenic mechanism. This causes an immunosuppressive TME that promotes immune suppression, preventing the fight against cancer (102). The efficacy of PDT is also reduced due to the TME-compressed tumor extracellular matrix, which impedes the infiltration of oxygen and chemical therapeutic species (23, 98). This is, therefore, suggestive of the importance of combination therapy, where PDT can be used with other forms of cancer therapy to surmount its therapeutic pitfalls. A PDT-optimized procedure in combination with IOT could produce an excellent synergistic impact against resistance in cancer treatment.

## 3 Cancer immunotherapy

Cancer IOT is a biological therapeutic modality that works by enhancing the immune system’s defenses to combat cancerous cells. The thymus, spleen, bone marrow, and lymph nodes are among the organs that constitute the immune system. Immune cells such as dendritic cells, B and T lymphocytes, monocytes, natural killer (NK) cells, and granulocytes, and signal proteins that highly include cytokines (INFα, TNFα, IL-11, IL-6, and IL-2) and chemokines (CXCL10 and CXCL9) help safeguard the host organism against cancer (15, 103). Active and passive IOT form the main categories of IOT, principally achieved by artificial stimulation of the adaptive and innate immune systems. Active IOT makes use of monoclonal antibodies [particularly the immune checkpoint inhibitors (ICIs)] or immunostimulation changes that occur during the release of cytokines. This category also includes IOT that directly modulates the immune response, like antigen-independent or antigen-dependent (for example, anticancer vaccines). Contrarily, passive IOT instead makes use of arginase inhibitors and small-molecule indoleamine 2,3-dioxygenase (IDO), along with genetically modified immune cells, including NK cells and T cells (adoptive therapy) (15, 104).

The immune system plays a vital role in combating cancer, and it may be broadly classified into two categories: the innate and adaptive immune systems. The system for the immune system is mostly made up of mucous membranes, epithelial barriers, neutrophils, macrophages, granulocytes, NK cells, and DCs (105). The system for the adaptive immune system mainly consists of humoral immunity, which principally consists of B cells, and cellular immunity, which is chiefly piloted by T cells (105, 106). The innate and adaptive immune systems should simultaneously function in the TME to effectively stimulate anticancer immunity. The main processes involved in stimulating an anticancer immunological effect can include (a) immature DCs recognizing cancerous cells or phagocytosing cancer-derived antigens (Ags), leading to their maturation. Also, through phagocytosis, the cancerous cells can be engulfed directly by macrophages (107); (b) fully developed DCs go through the lymphatic vessels to the lymph nodes, where they stimulate NK and T cells (108–110); (c) NK and T cells that are activated can migrate through the blood vessels to TME (111, 112); (d) cancer cell lysis is mediated by activated NK and T cells or macrophages. Nonetheless, the exhaustion of NK and T cells instead encourages tumor immune escape (113, 114).

### 3.1 Current-day’ cancer immunotherapy

Cancer IOT has greatly evolved and now has various therapeutic approaches that are designed to mimic the natural antitumor immunity of the body in order to combat cancer and extend the patient’s life (115). The different therapeutic approaches are outlined below include IOT vaccines, adoptive cell therapies, immune checkpoint inhibitors IOT, monoclonal antibodies IOT, and oncolytic virus therapy.

IOT vaccines: IOT vaccines for cancer treatment are developed to increase immune cells’ potential against cancer (116). They are divided into protein- and peptide-based vaccines (117), vector- and bacterial-based vaccines (118, 119), nucleic acid (RNA, DNA, self-amplifying RNAs (saRNA), and mRNA)-based vaccines (120–122), and cellular (DC and whole cell)-based vaccines (123).

Adoptive cell therapies (ACTs): In ACT, the NK and T cells or other cells from the patient are grown and expanded through engineering or without engineering, then infused into the patient to fight cancer (124, 125). The most common type of ACT is those derived from T-cells. The ACT based on T-cells can be accomplished by at least three unique T-cell approaches. Tumor-infiltrating-lymphocytes (TILs)-based ACT is the first approach, where endogenous TILs obtained from the patient tumors are grown *ex vivo* and injected into the patient (125, 126). ACT, based on the fabricated T-cell receptor (TCR), is the second approach. This type of ACT helps recognize particular tumor antigens; however, it is restricted to Ags expressed by the major histocompatibility complex (126). ACT based on chimeric antigen receptors (CARs) is the third approach. Chimeric receptors comprising a domain for recognizing extracellular antigens, a signaling domain in the cytoplasm, and a transmembrane domain are engineered with T cells obtained from the patient, producing CAR T-cells that lock onto and eradicate the specific types of cancer (127, 128).

Immune checkpoint inhibitors (ICIs): Immunotherapy ICIs derived medication can boost tumor cell immune-mediated clearance procedure and obstruct the co-inhibitory signal pathways to reactivate immunity against the tumor (129, 130). For example, some types of cancerous cells excessively secrete on their surface the programmed death-ligand (PD-L1) in order to avoid immune surveillance. This causes the activation of the “cytotoxicity brake” to continuously working, leading to T cell exhaustion and positive PD-L1 cancer cell survival (131). Also, PD-L1 and programmed cell death protein 1 (PD-1) (132), T-cell immunoglobulin mucin-3 (TIM-3) (133), lymphocyte activation gene 3 (LAG-3) (134, 135), and cytotoxic T-lymphocyte-associated antigen 4 (CTLA-4) (132) are frequently used immune checkpoint proteins.

Monoclonal antibodies (mAbs) IOT: mAbs are large-molecular-weight glycoproteins that are fabricated by B cells. They simulate induction of a durable antitumor response while also targeting cancerous cells directly (136). Three kinds of mAbs are commonly utilized in cancer treatment, which include the bispecific mAbs consisting of two distinct proteins attached together either covalently or non-covalently (137), antibody-drug conjugates, which are designed by conjugating mAbs with radioactive particles or chemotherapy drugs (138, 139), and naked mAbs (unconjugated mAbs) (140). However, there are several kinds of mAbs, which are linked to the nature of their heavy chain structures, and IgGs are currently the type most frequently utilized in antibody therapy (141, 142). Besides, the antibody (Ab) scaffold is a crucial component of tumor IOT. Findings from a recent study demonstrated that a VL one-domain antibody scaffold (rabbit-derived Ab scaffold) combined effectively with the drug 7-ethyl-10-hydroxycamptothecin (SN-38) to substantially hinder canine non-Hodgkin lymphoma (cNHL) cell *in vivo* and *in vitro* proliferation. This furnishes important theoretical justification for the application of Ab scaffolds in cancer IOT (143).

Oncolytic virus (OVs) therapy: OVs are one of the latest developments in cancer IOT (144, 145). OVs are a group of viruses that can be produced experimentally or exist naturally (146). OVs has the therapeutic ability to selectively replicate and spread in cancerous cells, destroying cancerous cells while avoiding damage to normal cells (147). In addition, OVs can be utilized for *in situ* vaccination (148), while immunological modulatory transgenes can be transferred to it and might even be utilized in conjunction with different therapies, such as chemotherapy and cell therapy (104, 149, 150). OVs are not limited to their application as oncolytic drugs; they can also serve as efficient carriers of anti-cancer genes and perform several functions at once, including gene therapy and virotherapy (151). The first OV medication authorized in 2015 by the U.S. FDA is T-vec (Talimogene laherparepvec, Imlygic) and is used for the treatment of recurrent melanoma in patients having topical non-resectable skin, lymph node, and subcutaneous lesions (152). Yet, the efficiency of OVs often depends on combination therapies, while stand-alone oncolytic virus treatments are subject to variation based on different factors, such as the immunological condition of the patient, the type of oncolytic viruses, and the kind of tumor. Moreover, current OVs exhibit poor infiltration and can be swiftly eliminated by antiviral responses (153).

For an effective cancer IOT, it might be essential to devise alternative methods to enhance immunogenicity and boost antitumor efficacy. Nonetheless, combinational therapies such as immuno-photodynamic therapy have emerged as a pivotal treatment strategy to address these shortcomings of mono-immunotherapy (154). Different research has demonstrated that PDT can stimulate an immunological response against tumors through an ICD mechanism. This type of treatment strategy is anticipated to compensate for the setbacks of each of the mono-therapeutic modalities and induce a healthy immune system to combat cancer. It may also offer stronger IOT immunogenicity as well as stop the proliferation of tumor cells that are still present in the body following PDT (102, 115).

### 3.2 Advantages and disadvantages of immunotherapy

Compared with conventional cancer therapy, radiotherapy, chemotherapy, and surgical intervention, IOT significantly increases patient survival by using various strategies, targets, and directives to combat cancer. IOT can target tumor tissues specifically while minimizing damage to normal tissues (155, 156). Two important causes of cancer death, metastasis and recurrence, could originate from cancerous cells developing defenses by evading immune surveillance (104, 157). About 90% of cancer-related fatalities worldwide are caused by metastatic malignancies, for which IOT has become a groundbreaking treatment option (18). IOT is unequivocally achieved using a variety of techniques to boost pre-existing immunity, reconstruct immune suppression within the TME to combat the targeted cancerous cells, and effectively trigger the innate and adaptive immunity. In improving surveillance and clearance function to stop cancerous cells from metastasis and recurrence, IOT not only amplifies the immune response in the initial tumor during therapy, but the therapy also excites systemic and long-lasting protective benefits (104, 158). Moreover, immune checkpoint blockade for IOT, such as PD-1 or PD-L1 inhibitors, has recently shown encouraging clinical results following treatment of patients with different types of cancer (159, 160). IOT methods such as cancer vaccines (161, 162), CAR T-cell therapy (163), and cytokine therapy (164) have evolved and have been proven to extend the progression-free survival of cancer patients and animal models under preclinical investigation. Also, to date, different advanced-stage cancers have been successfully treated using IOT, although different problems are encountered with its development (104, 165).

Solid tumor patients show a low response rate to IOT, which limits the therapy’s efficacy (166). Besides, first-generation immunotherapy-based cancer vaccines have had poor outcomes in clinical trials (167, 168). Immune-related side effects also hinder the effectiveness of treating cancer through IOT. Autoimmunity and immunological toxicity are becoming more widely recognized as significant clinical problems (104, 165). Also, conventional methods for IOT are unable to convert non-immunogenic (cold) cancer to immunogenic (hot) cancer (165, 169). “Hot” tumors are defined by an active immune response that frequently exhibits high levels of immune cell infiltration and responds well to immunotherapy, especially immune checkpoint blockade-based treatments. The “hot” tumors often exhibit an immune-inflamed property, with important infiltration of immune cells, especially CD8+ T cells (170). The TME of “hot” tumors is immunosupportive, which increases the effectiveness of immunotherapy, hence leading to better immune checkpoint blockade therapy in patients with “hot” tumors (171, 172). Conversely, “cold” tumors are non-immunogenic, immunosuppressed, and have inadequate T cell infiltration. The “cold” tumors can effectively camouflage themselves so as not to be recognized by the immune system, lowering the immune response efficiency and hence hindering antitumor therapy (102). Also, “cold” tumors are commonly described as an immune desert since they significantly lack immune cells (170). Therapeutically, cold tumors often respond poorly to most IOT immune checkpoint blockade treatments. However, some tumors may have both “hot” and “cold” tumor traits, making treatment options more difficult and requiring additional novel biomarkers and therapeutic combinations. Still, the “hot” and “cold” tumor categorization helps in understanding the therapeutic limitations and the responsiveness of cancer treatment (170, 173).

The limitations of current IOT techniques are linked to several factors that contribute to cancer’s overall resistance. Cancerous cells are developing resistance to IOT, leading to primary, adaptive, and acquired resistance, which greatly impedes cancer IOT (104, 165). For instance, failure can arise due to an immunosuppressive TME (consisting of stromal and cancer cells), which expedites tumor immune escape. Immunosuppressive molecules are secreted by stromal cells such as cancer-associated fibroblasts (CAFs) or tumor-associated macrophages (TAMs), which prevent cytotoxic T lymphocyte infiltration and stimulation, hence decreasing their potential to kill tumor cells. Also, the stromal surrounding components containing dense extracellular matrix and aberrant tumor vessels hinder effector T-lymphocyte infiltration into the tumor and enhance hypoxia. This consequently enhances immune suppression by distorting the production of cytokines, recruiting myeloid cells to suppress the immune system, and hampering cytotoxic T-lymphocytes’ killing action against cancerous cells. The aberrant tumor vasculature also controls solid tumors’ immune escape and restricts the distribution of immunotherapeutic molecules into the tumor (174). The efficacy of IOT is also limited since it does not work for all patients, and this is linked with low clinical response rates and solid tumors’ insufficient immunogenicity (175). Similarly, our body’s physiological and pathological barriers may obstruct the uptake of immunotherapeutic drugs or natives, making the bioavailability of the drugs considerably more challenging. In addition, certain types of cancerous tumors poorly react to IOT due to the absence of an immunogenic TME (176, 177). Furthermore, present-day clinical IOT is faced with the challenge of over-activated autoimmunity and inadequate immune response. For instance, IOT cancer vaccines are not used for all forms of cancer, as they can only induce the activation of the immune system in specific forms of cancer (178); immune suppression mediated by the tumor tissue can render ACTs to malfunction (179); ICIs may result in unfavorable organ damage perpetuated through the immune system (180); and mAb treatments may cause an overactive immune response such as cytokine-release syndrome (181, 182).

The alluded problems, therefore, hamper the advancement and wider execution of cancer IOT, and if unresolved, could lead to the reiteration of mistakes similar to those observed in some conventional cancer therapy (104, 165). However, combination cancer treatment strategies are strongly encouraged (183). The rationale behind this combination treatment seems to be based on the fact that molecularly targeted treatment can significantly impact the antitumor immunity to evoke a potential synergy if utilized with IOT. The combinational therapy approach may increase the efficacy and comprehensiveness of treatment by simultaneously targeting several tumor pathways. As a result, novel IOT approaches needed to circumvent tumor immune evasion are desperately needed.

## 4 Combination of PDT with IOT (photoimmunotherapy)

**Figure 3:**
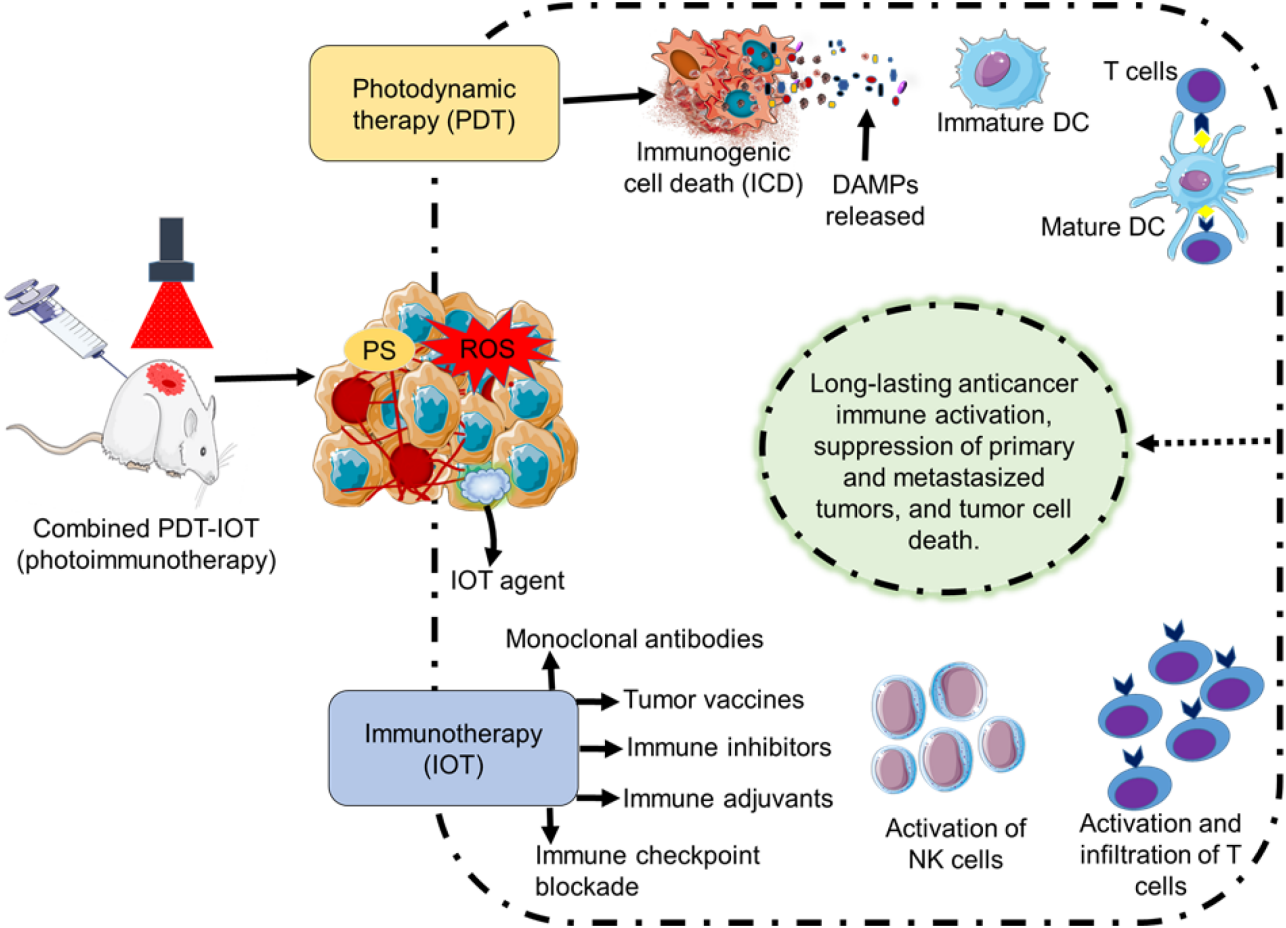
Illustration of photodynamic therapy (PDT) and immunotherapy (IOT) combined therapy (photoimmunotherapy) in preclinical studies. PDT can activate an immunological antitumor effect through the induction of an immunogenic cell death. IOT methods (monoclonal antibodies, immune inhibitors, immune adjuvants, immune checkpoint blockade, and tumor vaccines) in combination with PDT can elicit an immune response through the stimulation of NK cells and T cells. Overall, treatment by photoimmunotherapy can result in long-lasting tumor immunity, inhibition of the original and metastasized tumor, and eradication of tumor cells.

Combined treatment of PDT and IOT (photoimmunotherapy), can trigger both systemic and local immune responses in animal studies (preclinical studies), resulting in longer-lasting immune activity, more tumor cell death, and slower tumor growth (**Figure 3**). Among the notable advantages of this combination treatment are enhanced antigen presentation, reduced Tregs, increased T cell stimulation, and strengthened resistance to recurrence, regardless of the type of photosensitizer that is used. It is reported that treatment combining PDT and CpG oligodeoxynucleotide can reduce metastases, potentially amplify the activation of CD8+ T cells, and prolong survival (184, 185). Similarly, a two-stage treatment intended to target the tumor directly combined a low PDT immunogenic dose followed by an elevated dose was documented. Prolongs survival and slow growth of metastases tumor was noted, albeit the effects vary depending on the tumor cell line (58, 69). PDT-based immunotherapy is generally a versatile therapeutic method and is suitable for a wide range of patients since the treatment does not depend on a precise tolerance profile or genetic predisposition (69, 186). To achieve specific immunological stimulation in cancer treatment through photoimmunotherapy, different promising approaches involving monoclonal antibodies, immune inhibitors, immune adjuvants, immune checkpoint blockade, and tumor vaccines are used in preclinical settings to overcome tumor resistance and improve treatment outcomes.

### 4.1 Preclinical methods and evidence of photoimmunotherapy

#### 4.1.1 Photoimmunotherapy antibody-photosensitizer conjugate

To overcome anticancer treatment resistance and improve treatment efficacy, antibody-photosensitizer conjugates (AbPCs) have emerged as a viable strategy of combining PDT and targeted immunotherapy. The AbPCs are designed in such a way that the antibodies target the tumor tissue precisely, and the photosensitizer is also deposited in the tumor, which, when activated by light, induces ROS production that kills tumor cells. It is important to note that AbPCs both directly destroy cancerous cells and promote an immunological response against cancer (187, 188). This indicates that PDT’s selectivity and efficacy can be increased by conjugating PS with mAbs in the right way to activate an immunological response. Furthermore, the poor distribution of PS, because of the hydrophobic nature of most PSs, can be surmounted by incorporating PSs with specific antibodies. This helps to facilitate the internalization of the PS alongside the antibody (15, 188, 189). For instance, in a colorectal cancer model, a conjugate composed of chlorin e6 and cetuximab (cetuximab‐maleimide‐poly(ethylene oxide)‐poly(propylene oxide)‐poly(ethylene oxide)‐chlorine e6 conjugate, CMPXC) in PDT markedly elevated the cytotoxic T-cell and dendritic cell population (187). Likewise, PDT employing AbPCs can trigger the release of danger signals and antigens, strengthening anticancer immunological responses (16). Also, developed biomimetic photosensitizers [aggregation-induced emission (AIE) photosensitizers] with hitchhiking and antigen-presenting capacity (DC@AIEdots) stimulated T-cell proliferation and activation *in vivo*, stimulating the immune system (190). The conjugation of mAbs with aluminum phthalocyanine was validated. The low concentration of the free photosensitizer in the TME did not produce a significant PDT effect; however, conjugating the antibodies with the photosensitizer substantially elevates the selectivity and cytotoxicity effect of PDT while preserving the targeting molecule’s stability, integrity, and immunological reactivity (15, 191).

Likewise, by specifically binding to tumor-associated antigens, an antibody conjugated to PS enables effective delivery of the therapeutic agent to cancer cells, resulting in more efficient treatment (192, 193). For instance, the conjugation of antibody against epidermal growth factor receptors (EGFR) and the near-infrared (NIR) phthalocyanine dye IR700 has demonstrated encouraging outcomes in causing selective cell death following exposure to NIR light (194). Interestingly, photoimmunotherapy can provide a special benefit when it comes to drug resistance mechanisms, especially those involving drug efflux pumps. It is demonstrated that conventional PSs like hypericin interact with breast cancer resistance protein and multidrug resistance-associated protein-1, which are known to contribute to drug resistance (195). Photoimmunotherapy treatment can potentially bypass the drug efflux mechanism, guaranteeing adequate intracellular accumulation of the PS (194, 196). For instance, photoimmunotherapy is reported to successfully lower the survival of these resistant cells, improving the effectiveness of treatment as a whole by targeting cancer stem-like cells noted to display drug efflux mechanisms (192, 193).

Antibody and PS conjugation can be formed through different conjugation techniques, including genetic and chemical techniques that exploit specific functional groups like N-chlorosuccinimide (NCS), triazoles, and thiol. Frequently used chemical methods use reactive groups found on the photosensitizer and antibody, such as carbodiimide, isothiocyanate, or NHS (succinimidyl) ester, to produce conjugates (15, 188, 189). In genetic techniques, the antibody and the photosensitizer are fused directly using a recombinant protein, producing a single molecule (197–199). Using chemical techniques in bioconjugation enables the attachment of the PS to a specific area on the antibody. The bioconjugation approach for PDT and related therapeutic applications could be divided into different categories, including isothiocyanate conjugation, amide conjugation, tetrapyrrole-nanoparticle-antibody conjugates, maleimide conjugation, and copper-catalyzed azide–alkyne cycloaddition (CuAAC) reactions (“click” reaction) (15, 200–202). **Table 2** presents some of the conjugation methods involved in antibody-photosensitizer conjugation for targeting specific tumor.

**Table 2:** Conjugation methods involved in antibody-photosensitizer conjugation and their anticancer target.

Antibody	Photosensitizer	Conjugation method	Anticancer target	Ref.
Trastuzumab (α-HER2)	IRDye700	Formation of amide bonds with lysine residues through activated ester	Gastric carcinoma (NCI-N87)	(203)
Cetuximab (α-EGFR)	Benzoporphyin monoacid ring A	Formation of amide bonds with lysine residues through activated ester	Chinese hamster ovary cell line expressing EGFR (CHO-EGFR)	(204)
α-CD104	2,5-dioxopyrrolidin-1-yl 4 (4Z,10Z,14E,15Z,19Z)- 10,15,20-tri(pyridin-4-yl)-1H,21H-porphyrin-5- yl)benzoate	Formation of amide bonds with lysine residues through activated ester	Human bladder transitional cell carcinoma (UM-UC-3)	(205)
Trastuzumab (α-HER2)	(8S)-5-(carboxymethyl)-7-(3-carboxypropyl)-18-ethyl-2,8,12,17-tetramethyl-13-vinyl 7H,8Hporphyrin- 3-carboxylic acid	Formation of amide bonds with lysine residues through activated ester	Epithelial human breast cancer (MDA-MB-231), human breast cancer	(206)
HuHMFG1	Pyropheophorbide a	Formation of amide bonds with lysine residues through activated ester	Human colorectal adenocarcinoma (HT-29), human oesophageal adenocarcinoma (OE19)	(207)
LAG-3	5-(4-isothiocyanatophenyl)-10,15,20-tris-(4-N-methylpyridiniumyl) porphyrin trichloride	Isothiocyanate to amines on lysine residue	Human colorectal cancer (Caco-2)	(208)
α-EpCAM	4-(15-(4-isothiocyanatophenyl)porphyrin-5-yl)-1-methylpyridin-1-ium chloride	Isothiocyanate to amines on lysine residue	Human colorectal cancer (LoVo), Lung large cell carcinoma (CORL23)	(209)
35A7	5-(4-isothiocyanatophenyl)-10,15,20-tris-(4-N-methylpyridiniumyl) porphyrin trichloride	Isothiocyanate to amines on lysine residue	SKOv3-CEA-1B9 tumor	(210)
35A7 FSP 77	5,5′,5′’-(20-(4-isothiocyanatophenyl)porphyrin-5,10,15-triyl)tris (benzene-1,3-diol)	Isothiocyanate to amines on lysine residue	Colorectal adenocarcinoma (LS174T), Ovarian adenocarcinoma (SKOv3)	(210)
SIP (LI9)	4,4′,4′’-(20-(4-((1-(4-((2,5-dioxopyrrolidin-1-yl)methyl)cyclohexyl)-1-oxo-5,8,11,14,17,20,23-heptaoxa-2-azapentacosan-25-yl)carbamoyl)phenyl)porphyrin-5,10,15-triyl)tris(1-methylpyridin-1-ium)	Reduction via disulfide bridge and reconnection of free thiol using maleimide	LM fibroblasts, immortalized human embryonic kidney cells (HEK293T), Chinese hamster ovary cells (CHO-S), HEK293T	(211)
SIP (LI9)	4,4′,4′’-(20-(4-(2,5-dioxo-2,5-dihydro-1H-pyrrol-1-yl)phenyl)porphyrin-5,10,15-triyl)tris(1-methylpyridin-1-ium)	Reduction via disulfide bridge and reconnection of free thiol using maleimide	Vaccinia-infected mouse fibroblasts (LM fibroblasts), CHO-S, (HEK293T)	(211)
Trastuzumab (Fab)	Porphyrin	Reduction via disulfide bridge and reconnection of free thiols using maleimide propargylmaleimide proceeded by CuAAC (“click” reaction)	Invasive ductal breast carcinoma (BT-474), metastatic breast adenocarcinoma (MDA-MB-468)	(212)
Trastuzumab (IgG)	Porphyrin	Reduction via disulfide bridge and reconnection of free thiols using maleimide propargylmaleimide proceeded by “click” reaction.	MDA-MB-468	(213)

Isothiocyanate-derived conjugates are formed by joining the antibody’s amino group to the PS using the isothiocyanate active group (210). The LAG-3 antibody was fused with 5-(4-isothiocyanatophenyl)-10,15,20-tris-(4-N-methylpyridiniumyl) porphyrin trichloride through the isothiocyanate active group. The obtained conjugate demonstrated excellent PDT activity in the mouse model by significantly slowing the growth of the tumor. Also, the conjugate toxicity was noted to be tolerable in the animals tested (15).

Amide bioconjugations are a frequently used approach that assists in joining two molecules together through amide bond formation and are commonly executed by fusing activated esters with amines. For instance, the conjugation of the HuHMFG1 antibody with pyropheophorbide was reported. The conjugate was observed *in vivo* and *in vitro* in preclinical investigations to kill esophageal cancerous cells effectively. The conjugated compound thus shows promise in esophageal adenocarcinoma treatment and can provide a targeted and efficient therapeutic option for esophageal cancer (207).

Maleimide conjugation is the process of joining the antibody’s thiol group to the PS using the maleimide active functional group. To boost adaptability, maleimide functional groups could be integrated into bigger molecular weight linkers. For instance, the capacity of three types of porphyrins with a maleimide group linked to them to specifically mark cysteine residues on SIP-L19 antibodies [small immunoprotein (SIP)-antiangiogenic antibody (L19)] was studied. The antibody and photosensitizer complex was formed by the porphyrin’s aryl ring attaching directly to the antibodies or by utilizing the succinimidyl-4-(N-maleimidomethyl) Linker cyclohexane-1-carboxylic (SMCC). The SMCC was joined to the photosensitizer through polyethylene glycol (PEG) or an aliphatic chain. The obtained conjugate preserved the photosensitizer’s capacity to kill cells when activated by light and the antibody’s capacity to bind to its target (211). Another photoimmunotherapy photosensitizer was developed for the localized targeting and treatment of prostate cancer (PC) and PC stem-like cells (PCSC). The photosensitizer recombinant cysteine-modified anti-EpCAM and anti-CD44 antibodies conjugated with silicon phthalocyanine dye (WB692-CB2 dye) through a maleimide linker. The developed conjugate, after red light irradiation, exhibited target-specific binding and elevated cytotoxicity on PCSC and PC. The conjugate could serve in PC-efficient treatment while protecting the prostate gland and with reduced adverse effects. It could also be employed in radical prostatectomy to destroy residual cancerous cells or metastasized tumors in the lymph node areas in scenarios where surgery is infeasible (193).

In addition, the “click” conjugation technique is noted for being a very selective and effective chemical reaction and is gaining popularity in the synthetic chemistry sector. The CuAAC reaction (“click” reaction) is now documented to be commonly used in bioconjugation because of its selectivity, excellent yields, and biocompatibility (214). PDT photosensitizer-antibody bioconjugates developed using the “click” reaction method demonstrate high phototoxicity due to their enhanced optical ability in PDT and can selectively target and destroy cancerous cells (212, 213, 215). A combination of hydrophilic PEG and hydrophobic zinc phthalocyanine PS (C11Pc) stabilized water-soluble gold nanoparticles, which were then functionalized with jacalin (lectin or monoclonal antibodies specific to HER). The complex obtained in combination with PDT induced an enhanced production of singlet oxygen and phototoxicity in SK-BR-3 (breast adenocarcinoma cells) and HT-29 (colorectal adenocarcinoma cells) (216).

#### 4.1.2 Photoimmunotherapy immune inhibitors/immune adjuvants

Immunological adjuvants have been shown to be effective when used in conjunction with PDT. Imiquimod (a TLR7 agonist) is among the most promising adjuvants and has been approved by the FDA for the treatment of different skin diseases (217). Imiquimod causes DCs to mature and liberate pro-inflammatory cytokines by interacting with TLR7 on the endosomes and DCs (218). Imiquimod was used in a cream in combination with 5-aminolevulinic acid-mediated PDT to successfully treat skin squamous cell carcinoma (219). Also, it has been demonstrated that the artificial dipeptide called pidotimod can strengthen the immune response and guard against infection in humans and mice. However, the exact mode of action of pidotimod’s protective ability is unclear. According to a study exploring zebrafish models, PDT improved immune cell recruitment and enhanced the production of pro-inflammatory cytokines following the tail wound assay, yet protection from certain pathogen infections was not provided (220). PDT can also induce immunosuppression and inflammation because of contact hypersensitivity. Nonetheless, immune inhibitors are necessary to soften the immunosuppression signal of the tumor and allow for a fully induced PDT immune response (221, 222).

Anti-angiogenic peptides in combination with photofrin-mediated PDT preceded T cell activation and VEGF (vascular endothelial growth Factor) inhibition, resulting in improved PDT efficacy. An enhanced PDT impact and related immune response were equally observed when PDT was conducted in combination with granulocyte macrophage colony-stimulating factor (GM-CSF), IL-7, and IL-3. The GM-CSF and IL-7 were delivered into the tumor tissue by retroviral vectors, then PDT was conducted, followed by their administration. The T lymphocyte’s activity was stimulated by IL-7, and the macrophage maturation process was augmented thanks to GM-CSF activity. Recombinant cytokines, including IFN-γ, IL-1, IL-6, IL-8, and IL-18, were also utilized in conjunction with PDT. The efficacy of PDT combined with the cytokine-based therapy strongly relies on the timing and mode of their administration, correlated with the PDT procedure (223).

Also, PDT can be utilized in combination with nonspecific types of IOT, such as cytokine-based therapy. PDT in combination with an administered tumor necrosis factor alpha (TNFα) proves to boost the therapy’s efficacy. A TNFα antivascular inducer, 5,6-dimethylxanthenone-4-acetic acid (DMXAA), in combination with Photofrin-mediated PDT, occasioned a reduction in tumor volume and the resumption of a prolonged growth period in mice having the radiation-induced fibrosarcoma-1. This combinational treatment strategy led to tumor tissue necrosis with a reduction in blood flow and vascular density. The utilization of GM-CSF led to similar results by initiating the suppression of the growth of the tumor, prolonging the CT26 survival period, and bearing the LLC tumor in mice. An enlargement of anticancer immunity and complete tumor cell destruction was observed in 1/3 of the mice treated (58). Besides, protoporphyrin IX (PpIX)-mediated PDT treatment was enhanced with lipopolysaccharide (LPS). The method of combined treatment caused the decreased production of IL-6 and an elevation of IL-10 and TNFα levels (224).

#### 4.1.3 Immunotherapy/PDT vaccines

A promising type of combination therapeutic strategy is the utilization of PDT-treated cancerous cells to immunize DCs. Thus, PDT-induced anticancer vaccines are based on the anticancer immune response that is activated by PDT. In TME, PDT can activate an immune response that could be exploited to generate an anticancer vaccine (225). Cells of the immune system implicated in this type of response often include T-cells and DCs, among others. Studies based on PDT-generated vaccines revealed that T-cell stimulation produced long-lasting anti-cancer immunity (15). Korbelik, a renowned scientist, used PDT to create a vaccination for squamous cell carcinoma (SCCVII). He demonstrated that this vaccine inhibited the growth of tumors (226). The utilization of Par-ICG-Lipo filled with indocyanine green (ICG) for endoplasmic reticulum (ER)-targeted PDT induced ICD and demonstrated *in vivo* to improve the *in situ* tumor cells’ immunogenicity. This ER-targeted PDT, in conjunction with dendritic cells, may result in an effective clinical method for treating cancer by modifying cancerous cells into a vaccine for treating cancer (227). In another study, a dendritic cell vaccine containing glioma cells was stimulated by photosens-mediated PDT, resulting in ICD and the discovery of a four-gene signature linked to glioma patients’ general survival. This strategy may have the capacity to enhance the treatment of glioma by triggering Th17 immunity and helping in the prediction of patient outcomes (228).

Moreover, regulatory T-cells (Tregs), which are involved in hindering immunological activity against cancerous cells, can strongly affect the immunological response induced by PDT-generated vaccines. The depletion of these cell types can augment the efficiency of PDT-generated vaccines (229). The tumor whole-cell-derived vaccine can be optimized through interconnections with phagocytic receptors. The interconnection between phagocytic receptors and cancerous cells can enhance the effectiveness of PDT-generated vaccines. Scavenger receptors and phagocytic receptors (such as mannose receptors) are present on macrophages and dendritic cells. These receptors have the potential to recognize and attach to tumor-associated antigens, resulting in their retention and presentation to T-cells (230).

Also, tumor cell-derived vaccines can be optimized by using a different adjuvant like N-dihydrogalactochitosan (glycated chitosan) (231) or maneuvering cell death, especially that induced by necrosis (232). Dendritic cells can be activated by using adjuvants, consequently causing the adjuvant activation and thus robust production of an anti-cancer immune response for tumor-associated antigens expressing cancerous cells. Still, it is established that the activation of an immune response triggered by PDT-induced cell lysates on P815 and EMT6 cancerous cells does not need adjuvant co-administration (58). Similarly, research findings indicate that PDT-induced cell lysates activate DC maturation and the expression of IL-12 (233). A combination of PDT with immune checkpoint blockade or with immuno-regulatory activity dampening can also maximize the efficiency of PDT vaccines (15, 69). Other authors created a delivery nano drug system having doxorubicin hydrochloride and chlorin e6 (Ce6) as PS loaded on an amphipathic 4T1 breast cancer membrane that was coated by calcium carbonate. The concurrent action of PDT and PDT resulted in the liberation of tumor-associated antigens and ICD. It is anticipated that ROS produced by this technique will create PDT-DC vaccination by recruiting DCs through imitating inflammatory mechanisms (234).

#### 4.1.4 Photoimmunotherapy immune checkpoint blockade therapy

Immune checkpoint blockades (commonly monoclonal antibodies) are often used in IOT to target protein interactions that typically dampen the immune system (221, 235). The immune checkpoints exhibit undesirable immunomodulatory effects (236). Treatments based on immune checkpoint blockade are guided by helpful biomarkers such as tumor mutation burden. Future approaches could use tumor mutation burden and other biomarkers to better stratify individuals for IOT, which could help overcome the problem of high tumor burdens and poor treatment results, leading to cancer patients receiving an effective treatment (237–239). Current treatment strategies utilize target-specific antibodies to stop different immune checkpoints (235). The importance of PDT in such a treatment process is to promote tumor sensitivity and immunogenicity, thereby inducing ICD (221).

Medically used photosensitizers are being examined in conjunction with various immunotherapies targeting CTLA-4, VEGF, OX40 [tumor necrosis factor receptor superfamily member 4 (TNFRSF4)], EphA2 (ephrin type-A receptor 2), and immune checkpoint blockades, such as PD-1/PD-L1. The administration of antibodies that target PD-1 and its ligand, in conjunction with a vascular-targeted PDT (V-PDT) that utilizes Tookad^®^Soluble as a photosensitizer, modulated the immune system reaction, causing a high number of CD8+ T cells to infiltrate the TME. It also resulted in a decrease in the number and size of metastases and stimulated a general immune response (216). V-PDT in conjunction with OX40 and PD-1 targeted therapy also led to an elevated immune response (240, 241). Treatment using a conjugate composed of anti-EphA2 antibody and IRDye700 (near-IR fluorescent dye) promoted an increased ICD, conversely to treatment utilizing the photosensitizer alone (196). Immunotherapy that targets PD-L1 and CTLA-4 receptors in combination with PDT contributed to raising the survival rate of treated mice by activating various immune responses through inflammatory induction, phagocytosis, or improved leukocyte infiltration (242). An anti-VEGF therapy in combination with PDT improved antitumor response; yet, 24 hours after treatment, the build-up of the PDT photosensitizer [5,10,15,20-tetrakis(3-hydroxyphenyl) chlorin (mTHPC)] was reduced (243). Ripasudil, in conjunction with Ce6-embedded nanophotosensitizer-mediated PDT (FIC-PDT), triggered an ICD and incited the priming of tumor-specific cytotoxic T lymphocytes through the sensitization of antigen-presenting cells. This led to the activation of the PD-1/PD-L1 immune checkpoint blockade response, causing a strong antitumor impact in the melanoma intraocular model (244). PDT has been proposed as a possible complementary method to immune checkpoint inhibitors, including PD-1/PD-L1, CTLA-4, and CD47-targeted therapy. The principle of this treatment strategy is PDT’s potential to boost the immune system and strengthen the response to cancer induced by immune checkpoint inhibitors. PDT in conjunction with immune checkpoint inhibitors has produced good results in different studies, indicating the possibility of increasing the effectiveness of cancer IOT (221, 245). Immuno checkpoint blocking can be used to rewire the immune system to target the residual malignant cells after PDT has been used to target the original tumor in a multimodal treatment paradigm that combines IOT and PDT (241).

Moreover, PDT in conjunction with different therapies that focus on regulating macrophage activity or adjusting autophagy activity through chloroquine inhibition is being studied. PDT’s ability to activate a proapoptotic effect has increased due to its inhibitory effects on autophagic activity (246). The therapeutic outcome of PDT was enhanced by the macrophage-activating factor, achieving a 100% therapeutic efficacy. However, no alterations in tumor growth were perceived when the activating factor [D3-binding protein-derived macrophage-activating factor (DBPMAF)] was administered without PDT. The DBPMAF also lessened the immunosuppressive effect brought on by PDT (247).

Furthermore, transient hypoxia during PDT can change the phenotype of immune cells and tumors by upregulating PD-L1, which is reliant on hypoxia-inducible factor 1-alpha signaling. The possible changes linked with immune checkpoint homeostasis at post-PDT may work in concert with PDT’s stimulation of immune cell infiltration to support PD-1/PD-L1 blockage as a beneficial supplementary tactic (241, 248, 249).

#### 4.1.5 Challenges of photoimmunotherapy

Some patients might find the combined cancer treatment strategy, such as photoimmunotherapy, to be expensive and inaccessible, which can hinder the widespread utilization of the treatment (18, 250). A major obstacle to photoimmunotherapy platform development is the absence of clinical trial statistics to help forecast the photoimmunotherapy execution in humans. This could hinder photoimmunotherapy interventions from completely replacing traditional cancer treatment, yet photoimmunotherapy is anticipated to be more suitable and widely used as an adjunct therapy. Also, it is crucial to determine which patients stand to gain from this treatment approach (18).

Moreover, different perspectives, such as cancer resistance, immune-related adverse events (irAEs), toxicity, treatment parameter optimization, accessibility, and cost, can be examined to validate the setbacks and risks linked with PDT and IOT combined treatments. PDT uses photosensitizers, which can cause localized toxicity and negative consequences if they do not target precisely the required treatment area and can harm healthy tissues (18, 94, 251). Consequently, merging PDT and IOT may cause extreme toxicity, especially if the therapies are not adequately coordinated. However, irAEs that can include autoimmune responses may arise, where the body’s immune system targets healthy cells, which can be brought on by IOT. The irAEs may need to be managed carefully due to their severity (mild to severe effects). Also, treating the immune-related conditions could be complex, hence necessitating more interventions and medications, which puts an enormous burden on patients (18). Likewise, some IOT agents could induce an immunosuppressive TME that hampers the effects (dampening effect) of PDT-induced ICD (252). Additionally, PDT can worsen irAEs since inflammation brought on by PDT might cause adverse effects in both the treated area and nearby normal tissues, hindering treatment outcomes (253, 254). PDT-induced inflammation may cause or exacerbate adverse drug events such as myocardial damage and muscle weakness in individuals receiving immunotherapy, such as with tislelizumab (253). Also, PDT-induced inflammatory response may intensify the immunological activation brought on by PD-1 blockade, resulting in severe irAEs (254). Another major problem is figuring out the ideal parameters for exploring photoimmunotherapy, like dosage, treatment sequencing, and timing. Standardizing the treatment methods is challenging since every patient reacts differently. Cancer that is resistant to both PDT and IOT, and combining the two treatments, might still not be enough to get beyond the resistance mechanism (18). Nonetheless, more advanced and effective anticancer photoimmunotherapy methods using nanotechnology may use target-specific moieties for overcoming resistance and other treatment challenges.

## 5 Preclinical evidence of nano-photoimmunotherapy combating cancer resistance

Nanotechnology can improve photoimmunotherapy’s accuracy and effectiveness, providing answers to some of the treatment challenges. Nanomaterials with unique therapeutic attributes can exhibit high photoactivity, low toxicity, and multifunctional characteristics, and alongside a suitable wavelength of light stimulation, can deeply penetrate tumor tissues (18). Multiple treatment strategies can be successfully integrated into one platform thanks to the multi-functionality of nanoparticles (255, 256). Photoimmunotherapy assisted by nanotechnology methods is noted to present synergistic effects, improving the antitumor immunological responses (257). This indicates that major cancer treatment resistance phenomena that are linked with the TME, metastases, enhanced permeability and retention (EPR), and non-specific targeting may be addressed by incorporating formulated specific forms of nanoparticles in photommunotherapy treatment. This can help to overcome irAEs, toxicity, and treatment resistance.

### 5.1 Nano-photoimmunotherapy in specific tumor-targeting

Tumor-targeting through nano-photoimmunotherapy can minimize systemic toxicity, safeguard normal healthy cells, and strengthen photoimmunotherapy. Besides, the targeted delivery of immune modulators and photosensitizers into the tumor tissue through nanoparticles can augment the treatment effectiveness (258, 259). For instance, Deng et al. produced a reduction-sensitive nanocarriers (Ds-sP NPs) [PEG-s-s-1,2-distearoyl-sn-glycero-3-phosphoethanolamine-N-(amino (polyethylene glycol)-2000] that were successfully loaded with an endoplasmic reticulum ER-targeting PS (TCPP-TER) [4,4′,4″,4′″-(porphyrin-5,10,15,20-tetrayl)tetrakis(N-(2-((4methylphenyl)sulfonamido) ethyl)benzamide]. The obtained Ds-sP/TCPP-TER NPs complex exhibited a selective ER accumulation, resulting in local production of ROS, following treatment with the near-infrared laser. This induced an increased level of oxidative stress in the tumor cell ER, activating the DAMPs and hence producing an amplified ICD (260). Similarly, PDT combined with epidermal growth factor receptor blockade was investigated, where a nanobody-IRDye700 conjugate composite was administered to a mouse orthotopic tumor model. The results prove that the nanobody-PS conjugate composite accumulated in the tumor tissue, causing necrosis with a non-toxic impact on normal tissues (261). In addition, Hanaoka et al. developed a photoimmunotherapy photosensitizer nano-conjugate composite (drug), IR700-YP7, to target Glypican-3 (GPC3). Glypican-3 (GPC3) is a surface biomarker expressed on HCC cells and is a therapeutically attractive target since its expression is predominantly high in hepatocellular carcinoma (HCC) and not in healthy cells. Also, HCC is a deadly malignancy worldwide, and only a small percentage of patients with HCC can benefit from curative surgery (262, 263). The IR700-YP7 (IRDye700DX conjugated with anti-GPC3 antibody) photoimmunotherapy drug with nab-paclitaxel was used to treat A431/G1 tumors in mice. When the treated tumor tissue was exposed to near-infrared light treatment, the IR700-YP7 rapidly caused A431/G1 cell death. Photoimmunotherapy helped in decreasing the tumor growth in comparison with the untreated tumor and also facilitated the improved delivery of nab paclitaxel, hence boosting the treatment therapeutically (262). This is supported by the fact that AbPCs do not affect non-expressing cells, but they are only effective as a therapeutic drug when attached to the targeted cell membrane. Also, early after photoimmunotherapy, tumor vessels are not damaged and are permeable, promoting a dramatic increase in blood flow. This makes it easy for nanosized drugs of high concentration to be delivered to the specific tumor during treatment, with little uptake in the non-tumor targeted areas (264). In another study, HCC was targeted through its cell surface biomarker called EpCAM (epithelial cell adhesion molecule) (265). EpCAM plays a vital role in cell proliferation, adhesion, stemness, and migration. This makes EpCAM a possible IOT target in cancer treatment and is also useful as a prognostic marker and in diagnosis (266, 267). EpCAM has been reported to be a stem cell marker and greatly promotes the survival and metastasis of cancerous tumors, including HCC (267, 268). An anti-EpCAM-conjugated nano-micelle (anti-EpCAM-UPGs-MX) was developed by Han et al. An excellent EpCAM targeting signal was observed in HCC-bearing mice after they were treated with the conjugate. This was confirmed through a vibrant green fluorescence signal from the treated mice. The anti-EpCAM-conjugated nano-micelle also exhibited both passive and active targeting potential, leading to its elevated aggregation rate in the tumor even 48 hours after treatment. Conversely, the untargeted micelles exhibited passive targeting and no active targeting (265).

In clinical practice, targeting tumors through the EPR effect does not always result in fruitful outcomes, as the EPR effect would rely on the tumor type and location, the macromolecular antitumor drug’s physical-chemical attributes, and the tumor blood perfusion state. However, nanoparticles can promote the EPR effect, making it possible to deliver the antitumor agent precisely. This can lead to increased accumulation of the antitumor agent at the tumor site and enhance blood supply to the tumor (269, 270). A study by Sano et al. developed a novel antibody conjugate consisting of an antibody and photosensitizer [RDye 700DX (IR700)] to enhance photo-immunotherapy by the EPR effect. The antibody conjugate could deliver nanoparticles (with sizes 10–200 nm) effectively into the tumor site. Highly selective cell killing occurred rapidly following 690 nm of phototreatment. The antibody bound maximally to the cells in the perivascular tumor space and caused the fast killing of tumor cells. When these cells are killed fast, vascular permeability rises, permitting rapid leakage of nanoparticles into the tumor. As a result, the photo-immunotherapy-treated tumor accumulated the nanoparticles up to 24 times above that of the control tumor, a phenomenon referred to as “super-enhanced permeability and retention.” Also, the photo-immunotherapy combined with liposome-daunorubicin treatment resulted in improved treatment and prolonged survival of the tumor-bearing mice (264). In addition, a nano-redox-activatable liposome (RAL) was developed and encapsulated with an indoleamine 2,3-dioxygenase (IDO) inhibitor (IDO@RAL). The RAL demonstrated an EPR effect by its increased tumor accumulation and prolonged blood circulation in mice with 4T1 tumors. If endocytosis occurred following treatment of the tumor, the nanovesicle may cause an exponential increase in PDT activity (>100-fold) and fluorescence signal due to the high glutathione threshold in the tumor intracellular space. Consequently, phototoxicity to healthy cells will be reduced, and tumor growth inhibition achieved thanks to the nano-activatable design. Interestingly, the RAL-mediated PDT led to cytotoxic T cells’ intratumoral infiltration by triggering the tumor cells in ICD. The treatment, in combination with the IDO inhibitor, led to an augmented systemic antitumor immunologic effect (271).

### 5.2 Nano-photoimmunotherapy in overcoming tumor microenvironment setbacks

Solid tumors commonly display certain TME resistance characteristics, such as extreme hypoxia, low pH, and elevated levels of glutathione (GSH) content, compared to healthy tissues. The TME can be modulated using nanoparticles to make it less immunosuppressive and more favorable for treatment that could trigger an immune response activation. Also, smart nanotechnology TME-sensitive components or chemical linkers can be designed to overcome the TME setbacks. TME-sensitive nanoparticle components, and also advanced nanosized metal–organic frameworks can efficiently escape the aggregation-caused quenching. This allows for photosensitizers to be released quickly in the tumor, leading to great elevations in ROS production (99, 258). The elevated ROS can strengthen the immunological response by boosting the effect of ICD. In addition, TME-sensitive nanoparticles can program the pharmacokinetics and location of both immunomodulators and photosensitizers smartly. This increases the tumor-targeting ability, resulting in optimized photoimmunotherapy without producing any acute side effects (99). For instance, a study by Zhen et al. developed a nano-photoimmunotherapy (nanoparticle-based photoimmunotherapy) to modulate the TEM for effective immunity against cancer. In the study, carcinoma-associated fibroblasts (CAFs) were combated by targeting a fibroblast-activation protein (FAP), noted to be highly expressed on CAFs’ surface (272). CAFs, as a major TME component, in both the original and metastatic tumors, can strongly affect the behavior of cancerous cells and exhibit multi-functions in tumor development, metastasis, angiogenesis, cancer stemness, metabolism, immunosuppression, and tumorigenesis (273–275). FAP is regarded as a universal antigen for tumor targeting, and its expression by multipotent bone marrow stem cells has been reported by different studies (272, 276, 277). A nano-photoimmunotherapy drug developed using ferritin (a solid nanoparticle-protein cage, serving as a photosensitizer carrier) was conjugated with FAP-scFv (FAP-specific single-chain variable fragment). The photo-irradiation treatment facilitated the nano-photoimmunotherapy drug to eradicate the CAFs in tumors, yet with little injury to the normal tissues because of the treatment location. Importantly, the nano-photoimmunotherapy resulted in strong suppression of the tumor in immunocompetent mice. Additional investigation revealed that the nano-photoimmunotherapy promoted a decrease in the secretion of C–X–C motif chemokine ligand 12 (CXCL12) and the deposition in the tumor extracellular matrix, which are all controlled in untreated tumors by CAFs and also regulate T cell exclusion, which stops T cells from direct contact with cancerous cells. CAFs’ selective killing through nano-photoimmunotherapy leads to substantial T cell infiltration, accompanied by effective suppression of the tumor (272). Another study serendipitously discovered that pH-responsive nanovesicles (pRNVs) (which are self-assembled from block copolymer polyethylene glycol-b-cationic polypeptide) are capable of more than just acting as nanocarriers, as the pRNVs also trigger ICD through exposing calreticulin on the surface of preapoptotic cells. The pRNVs composite was developed by encapsulating with indoximod (IND) (an indoleamine 2,3-dioxygenase inhibitor) and the photosensitizer [2-(1-hexyloxyethyl)-2-devinyl pyropheophorbide-a (HPPH)], forming a pRNVs/HPPH/IND composite. A low dose of pRNVs/HPPH/IND stimulated significant anticancer impact, and the photo-irradiation led to an abscopal effect on the B16F10 melanoma model. The major outcome of the treatment includes the generation of singlet oxygen through HPPH-mediated PDT, increased recruitment of DC and immunological response following ICD stimulation by PDT and pRNVs, as well as the modulation of TME by IND, which was up-regulated by P-S6K phosphorylation, leading to the inhibition of Tregs and the enlargement of CD8+ T cells. The study thus presents an “all-in-one” nanocarrier that uses multifunctional materials to enhance the effectiveness of cancer immunotherapy (278).

Tumor hypoxia remains a significant barrier to immunotherapy, PDT, and other cancer treatment methods, resulting in poor clinical prognosis (279, 280). Hypoxia is essential for developing an immunosuppressive TEM, since it controls programmed death ligand 1 (PD-L1) expression and immunosuppressive TAMs infiltration (281). However, nanocarriers can deliver therapeutic agents to reduce hypoxia, which is often common in TMEs, improving the efficacy of cancer therapy. To overcome hypoxia and enhance tumor treatment, oxygen-generating or oxygen-carrying strategies mediated by nanoparticles can promote oxygen elevation in the tumor tissue (279, 280). A core-shell nanoformulation (AuNC@MnO_2_, AM) was created in another study, consisting of a hollow gold nanocage (AuNCs) formulation covered with a manganese dioxide coat (282) for treating metastatic triple-negative breast cancer (mTNBC) through PDT-induced oxygen-boosted immunogenicity. mTNBC is a very aggressive form of cancer that is typified by producing elevated fatality and poor prognosis, even with systemic chemotherapy and radiotherapy interventions. The AuNC@MnO_2_, AM nanomaterial acts like a TME-responsive oxygen producer. The PDT-induced oxygen-boosted immunogenicity was stimulated thanks to the generation of ROS after the NIR irradiation treatment. In the presence of excessive H_2_O_2_ and in an acidic microenvironment, the manganese dioxide (MnO_2_) undergoes a reaction: MnO_2_ + H_2_O_2_ + 2H^+^ → Mn²^+^+ 2H_2_O + O_2_↑ in the tumor tissue, producing a lot of oxygen to enhance the build-up of ROS in the tumor tissue, while also increasing the effectiveness of PDT. This procedure elicits an ICD and the release of DAMPs, thereby inducing DC maturation and the stimulation of effector cells. This evokes a strong systemic immunological response against mTNBC. Also, the produced Mn²^+^ and oxygen are useful for multimodal imaging since they can generate fluorescence (FL)/photoacoustic (PA)/magnetic resonance, hence providing the possibility for integrating the diagnosis and treatment of tumors (282).

Another study described an MnO_2_-containing albumin nano-formulation for enhancing IOT through immunosuppressive TME modulation and tumor hypoxia alleviation. The MnO_2_-containing albumin nano-formulation facilitated the collaborative delivery of paclitaxel dimer, NLG919, and IR780 to augment photoimmunotherapy. An increase in oxygen supply was catalyzed by MnO2, promoting an effective paclitaxel-mediated therapy and PDT, which collectively enhanced the development of specific cytotoxic T cells and ICD. Interestingly, the increased oxygen supply relieves the tumor tissue from hypoxia, hence modifying the immunosuppressive TME by suppressing the PD-L1 expression and the M2-type TAMs infiltration in the tumor tissue. This enhanced the effectiveness and infiltration of cytotoxic T cells when combined with immune checkpoint blockade through NLG919, leading to the complete eradication of the primary tumor and almost completely halting tumor cell metastasis and relapse. This study validates a strategic therapeutic method for breast cancer by strengthening IOT through hypoxia relief by immunosuppression modulation and ICD induction (281).

### 5.3 Nano-photoimmunotherapy in addressing metastasis and recurrence

Cancer metastasis and recurrence remain the inevitable problems despite the utilization of robust treatment methods. Also, addressing the seeding and colonization of metastatic tumor cells remains difficult. The current IOT method seems to be more suitable for solving these problems. However, the clinical utilization of IOT is hindered by poor tumor antigen presentation, tumor tissue heterogeneity, and inadequate targeting (283, 284). Combination photo-immunotherapy procedures that use nanoparticles are encouraged, as they could enhance responsiveness in patients responding to cancer treatments (258). A study by Xu et al., 2017 engineered upconversion nanoparticles (UCNPs) that were concurrently filled with imiquimod (R837) (toll-like-receptor-7 agonist) and chlorin e6 (Ce6), producing a UCNP-Ce6-R837 complex that was used for preventing the recurrence of colorectal cancer by targeting both the primary and metastasized tumor. The UCNP-Ce6-R837 treatment, followed by near-infrared (NIR) irradiation, would lead to depth penetration of the tissue, leading to effective tumor destruction by PDT. This promotes the generation of tumor-associated antigens, which, when combined with the adjuvant (R837) containing nanoparticle (UCNP-Ce6-R837), can stimulate a robust anticancer immunological response. Interestingly, UCNP-Ce6-R837 plus PDT treatment merged with checkpoint blockade (CTLA-4) not only exhibited outstanding effectiveness in eradicating the tumor when subjected to NIR irradiation but also caused a potent anticancer immunity, preventing the development of the distant tumor that remained following PDT treatment. Moreover, the long-term immunological memory capacity of such an IOT method against cancer shields the treated mice against cancer cell reactivation (285).

Besides, a nano-chimeric peptide composite (nano-PpIX-1MT) was developed to target lung cancer tumors and metastasized tumors by photoimmunotherapy. The nano-PpIX-1MT integrates an immune checkpoint inhibitor [1-methyl-tryptophan (1MT)] with the photosensitizer [protoporphyrin IX (PpIX)] using a caspase-responsive peptide sequence [Asp-Glu-Val-Asp (DEVD)]. The nano-PpIX-1MT infiltrated the tumor site by an enhanced penetration and retention effect, followed by photo-irradiation treatment at 630 nm. This resulted in ROS production, which caused the cancerous cells to undergo apoptosis. This facilitated caspase-3 up-regulation and a strong immune response against tumor antigen production. The cleavage of caspase-3 was followed by 1MT release, subsequently enhancing the immune system and aiding in the efficient activation of CD8+ T cells. The nano-PpIX-1MT was therefore able to induce a cascaded synergic photoimmunotherapy effect by inhibiting the original tumor and metastasized lung cancer (283).

Furthermore, Ce6/BMS-202 NPs [Ce6/BMS-202/Bristol-Myers Squibb nanoparticles (NPs)] were prepared for synergistic PDT and IOT on 4T1 tumors. The NPs present beneficial properties, such as exhibiting a 100% drug loading ability and nontoxic and hydrophilic properties. BMS-202 (N-{2-[({2-Methoxy-6-[(2-Methyl[1,1’-Biphenyl]-3-Yl)methoxy]pyridin-3-Yl}methyl)amino]ethyl}acetamide) is a nonpeptidic small molecule capable of strongly inhibiting PD-1/PD-L1 interaction. Treating 4T1 tumor-bearing mice with BMS-202 NPs resulted in a significant decrease in 4T1 tumor growth, which was identical to the antitumor effect induced by anti-PD-L1 monoclonal antibody (α-PD-L1) treatment. Using Ce6 NPs in tandem with α-PD-L1 or BMS-202 NPs constantly leads to more anticancer and antimetastatic efficacy. This was accompanied by increased maturation of dendritic cells and improvement in antigen-specific T cells infiltrating the tumor tissue, hence resulting in over 90% of the original and distant tumors being inhibited. Also, the BMS-202 NPs can combat lung cancer metastasis and stop the recurrence of the tumor by providing immune-memory protection. BMS-202 NPs could possibly be used to replace monoclonal antibodies for cancer IOT applications, as antibodies present various therapeutic limitations, such as poor immunogenicity, ineffective tumor tissue penetration, and being very costly (286).

Similarly, the study by Guo et al. utilized nano-core-shell magnetic composites (MNCs) to develop an oxygen-independent photosensitizer for the treatment of triple-negative breast cancer models. The MNCs-mediated PDT treatment led to persistent production of free radicals by promoting the polarization of macrophages into pro-inflammatory M1 phenotype, electron-hole dissociation efficacy, and stimulating a systemic immunological response against the tumor (287) However, concurrent adaptive immune resistance was observed with the MNCs-mediated PDT treatment, which was typified by increased expression of PD-L1 on tumor tissue, macrophages, and DCs. The MNCs mediated PDT treatment in combination with checkpoint blockade significantly suppressed the original and metastasized tumors through three intervention processes ‘trident modality,’ which includes immunosuppressive TEM modification with inhibition of PD-L1 blockade and immunosuppressive cells; increased tumor-infiltrating-lymphocyte (TIL) rates; and steady generation of free radicals in both the hypoxic and normoxic states to directly eradicate the tumor. Likewise, the possible mechanisms responsible for metastasis inhibition were investigated using the lung tissue transcriptome expression profiling. The outcome indicated that the ‘trident modality’ modified several genes that are linked with cancer-related signal pathways and immune activation. This “trident modality” could be used widely in clinical settings and serve as a potential therapeutic strategy for managing cancer that is resistant to treatment (287). Moreover, mesoporous hexagonal core-shell zinc porphyrin-silica nanomaterial (MPSNs) with the ability to serve as a superior photosensitizer in photo-immunotherapy and also as a drug carrier to achieve a synergistic effect were loaded with R837 (imiquimod, a toll-like receptor-7 agonist) (MPSNs@R837) to stimulate photothermal therapy (PTT) and PDT ICD. This strategy led to strong immunological responses specific to 4T1 tumors in mice, by promoting dendritic cell maturation following the pH-responsive release of R837 and subsequently causing little toxicity and a strong suppression of both primary and metastatic tumors when in conjunction with the programmed death ligand-1 (PD-L1) checkpoint blockade. This treatment strategy thus demonstrates that the utilization of checkpoint blockade alongside PTT and PDT treatment can inhibit cancer metastasis (288).

## 6 Conclusion and perspective

Cancer PDT and IOT are promising therapeutic strategies. However, these treatment strategies are still ineffective in eradicating cancer due to their inability to surmount therapeutic defeats linked with cancer resistance. The involvement of PDT in activating anticancer immunological responses is highlighted in different studies. PDT is a well-liked treatment that has become a great way to enhance immunotherapies for a more effective cancer treatment. Various methods of incorporating PDT with IOT explore immunologic adjuvants, developed DC vaccines, immune checkpoint blockades, and antibody-photosensitizer conjugates and have led to excellent synergistic effects in preclinical studies. Still, the development of photoimmunotherapy seems to be in its early stages, since it seems to lack clinical evidence to fully validate its anticancer therapeutic potential in clinical settings. Major treatment setbacks, such as toxicity, treatment accessibility, and cost, also hamper photoimmunotherapy. Nanotechnology can help address some of the therapeutic issues by increasing the precision and efficacy of photoimmunotherapy. The amalgamation of tailored nanoparticles with photoimmunotherapy (nano-photoimmunotherapy) molecules or methods is confirmed in preclinical studies to offer cancer-targeted therapy, combat cancer metastasis and recurrence, and prevent resistance from the TME. This helps to reduce treatment systemic toxicity and overall cancer resistance while also promoting a long-lasting immunological anticancer response. Unfortunately, these promising outcomes are obtained from preclinical research conducted in a variety of *in vitro* and *in vivo* studies.

Photoimmunotherapy or nano-photoimmunotherapy clinical applications are strongly needed in order to fully warrant the efficacy of these combination therapies in eradicating resistant cancer in patients. To fully comprehend these anticancer therapeutic methods’ potential in clinical settings and further improve them, more research is necessary. Therefore, further clinical research or multiple clinical trial studies are solicited to determine the broader efficacy of photoimmunotherapy or nano-photoimmunotherapy procedures across diverse patient tumor types. This could lead to clinical advanced photoimmunotherapy molecules for different cancers being introduced to the market. This could help in overcoming cancer resistance in cancer patients.
